# Supplementary material for: A general rule on the organization of biodiversity in Earth’s biogeographical regions
Source: Nat Ecol Evol. 2025 Jun 4;9(7):1193–204. doi: 10.1038/s41559-025-02724-5 (PMC12240819; doi:10.1038/s41559-025-02724-5)
Supplement: Supplementary file 1 — Supplementary Figs. 1–35, Tables 1–12 and Appendices A–E. [file 41559_2025_2724_MOESM1_ESM.pdf]

nature ecology & evolution

Supplementary information https://doi.org/10.1038/s41559-025-02724-5

A general rule on the organization of biodiversity in Earth's biogeographical regions

In the format provided by the authors and unedited

Table-of-contents

Supplementary Figures 1-35

Supplementary Tables 1-12

Appendices A-E

References

Other Supplementary Information for this manuscript includes:

Supplementary Code: R code for calculating the biodiversity aspects and identify biogeographical sectors.

Supplementary Fig. 1 | Spatial distribution patterns of the four-biodiversity metrics in global biogeographical regions of amphibians. See biogeographical regions in Extended Data Fig. 2. See correlation values of the four metrics in Extended Data Table 2.

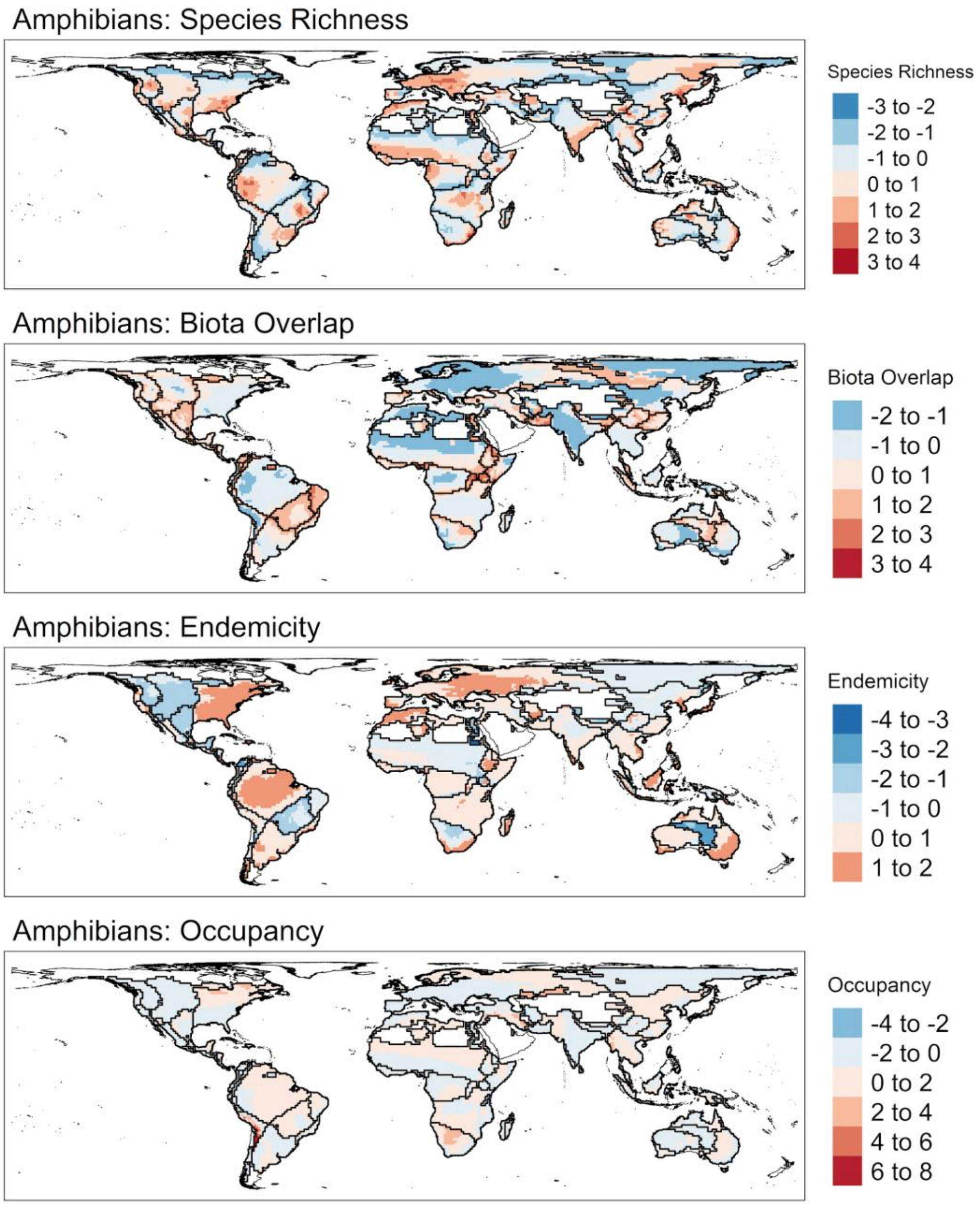

Supplementary Fig. 2 | Spatial distribution patterns of the four-biodiversity metrics in global biogeographical regions of birds. See biogeographical regions in Extended Data Fig. 3. See correlation values of the four metrics in Extended Data Table 3.

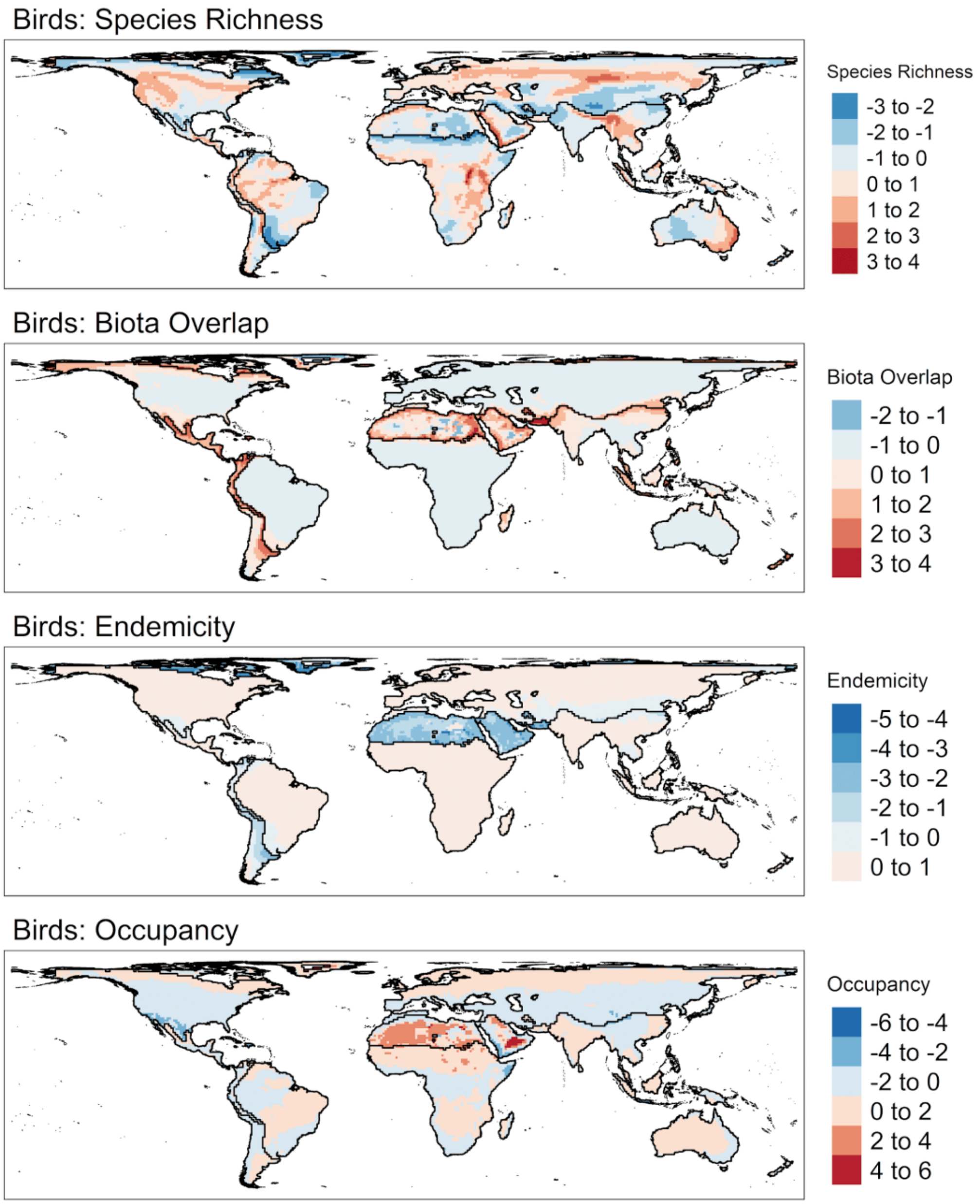

Supplementary Fig. 3 | Spatial distribution patterns of the four-biodiversity metrics in global biogeographical regions of dragonflies. See biogeographical regions in Extended Data Fig. 4. See correlation values of the four metrics in Extended Data Table 4.

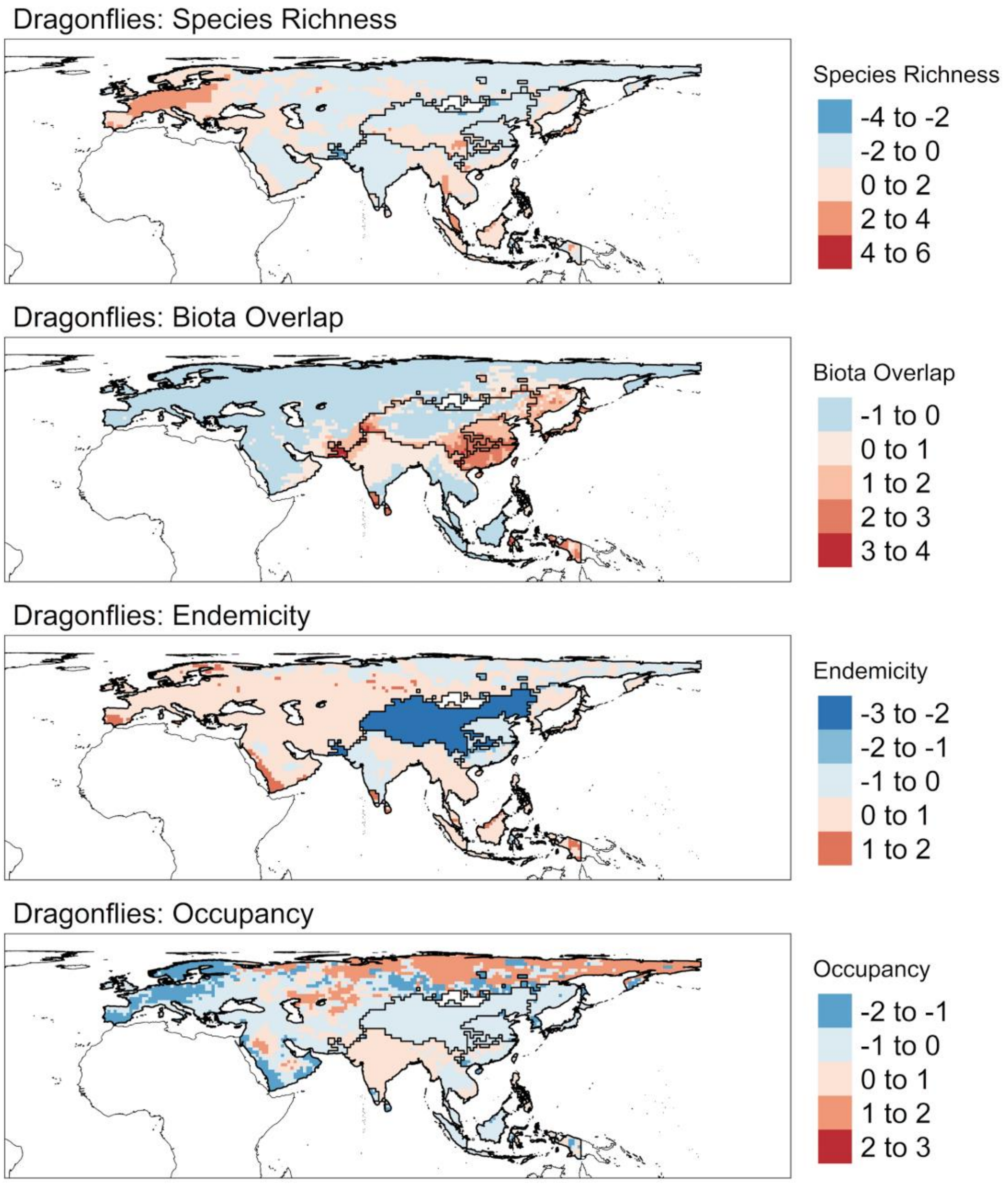

Supplementary Fig. 4 | Spatial distribution patterns of the four-biodiversity metrics in global biogeographical regions of mammals. See biogeographical regions in Extended Data Fig. 5. See correlation values of the four metrics in Extended Data Table 5.

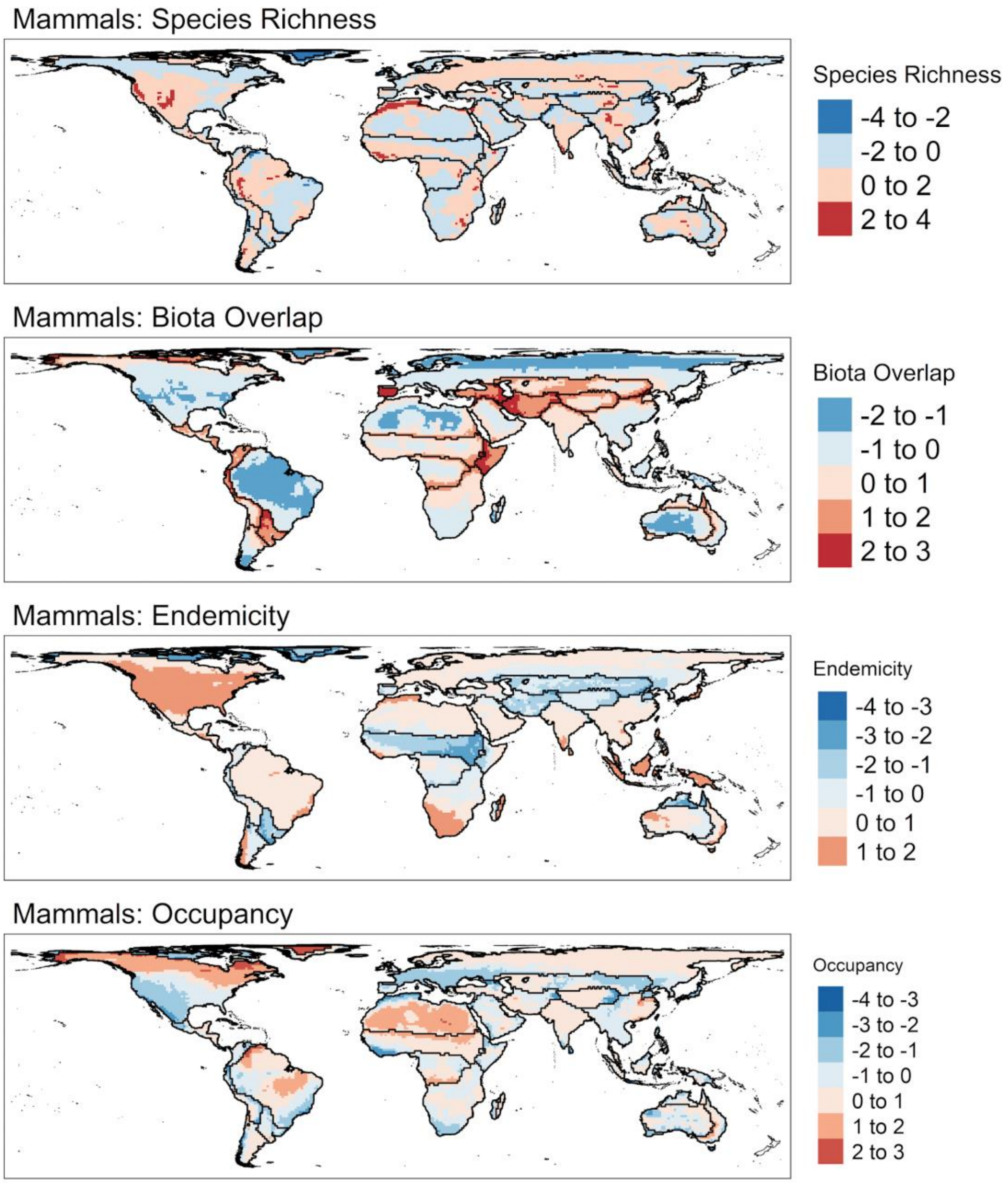

Supplementary Fig. 5 | Spatial distribution patterns of the four-biodiversity metrics in global biogeographical regions of rays. See biogeographical regions in Extended Data Fig. 6. See correlation values of the four metrics in Extended Data Table 6.

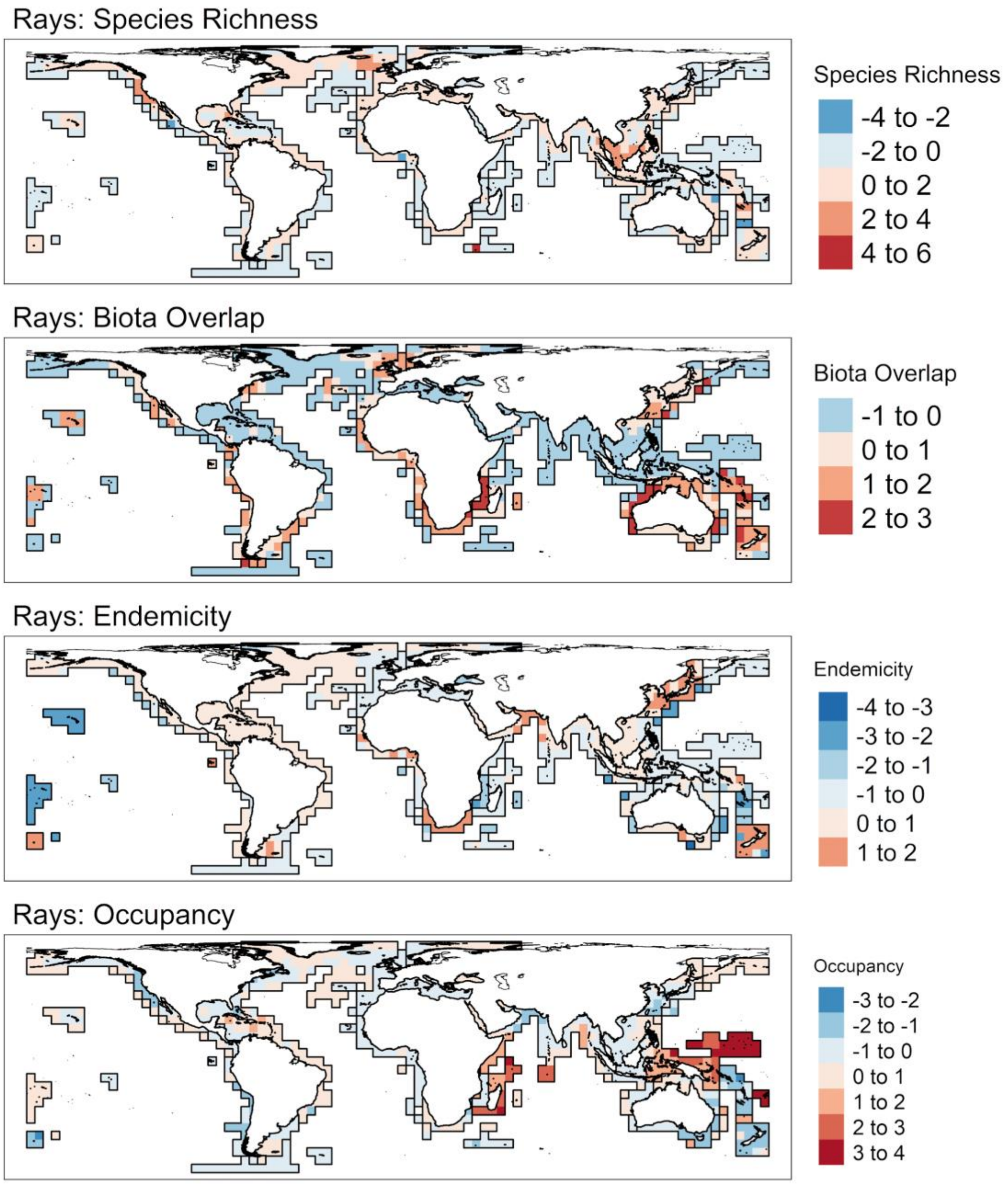

Supplementary Fig. 6 | Spatial distribution patterns of the four-biodiversity metrics in global biogeographical regions of reptiles. See biogeographical regions in Extended Data Fig. 7. See correlation values of the four metrics in Extended Data Table 7.

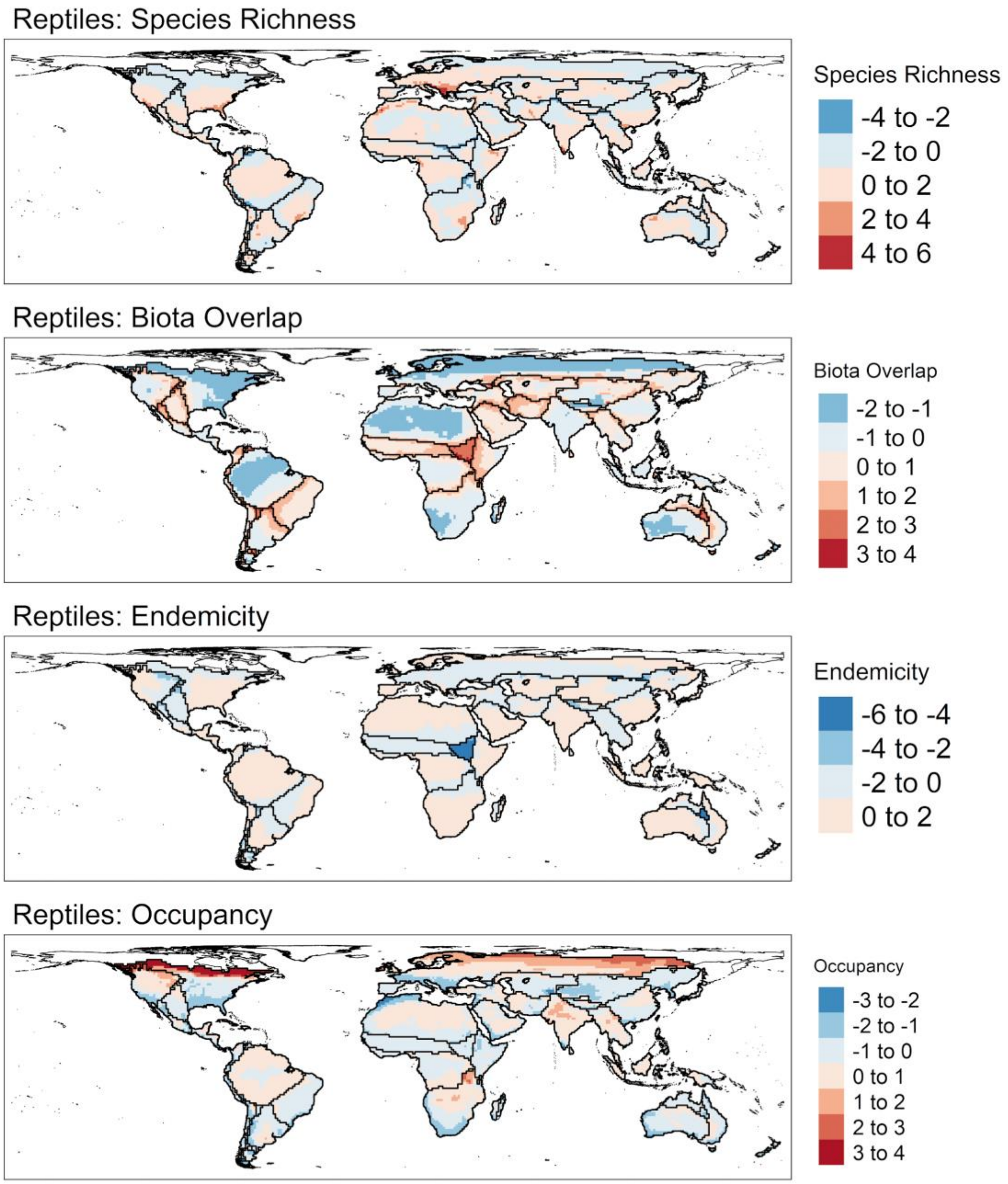

Supplementary Fig. 7 | Spatial distribution patterns of the four-biodiversity metrics in global biogeographical regions of trees. See biogeographical regions in Extended Data Fig. 8. See correlation values of the four metrics in Extended Data Table 8.

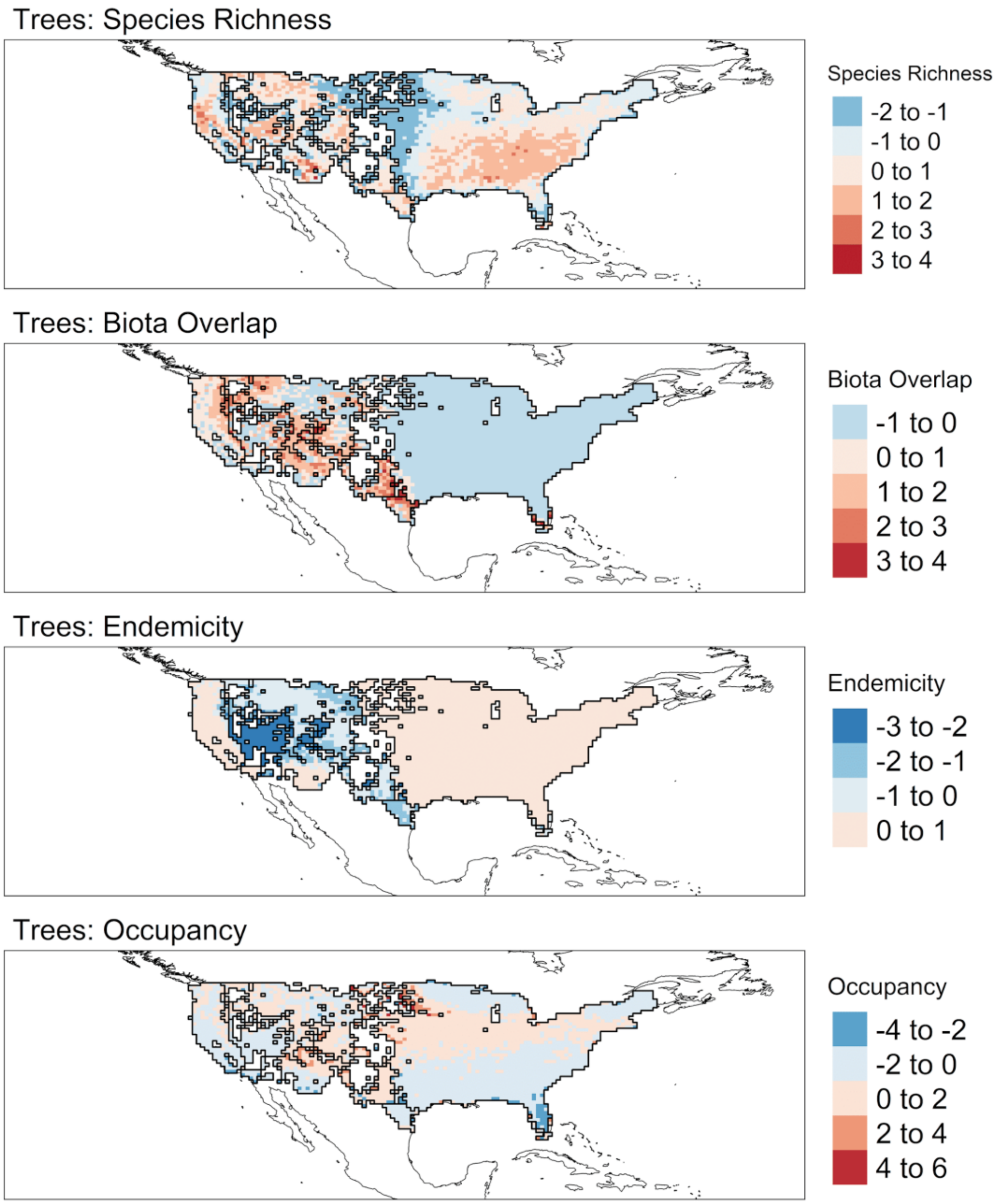

Supplementary Fig. 8 | Distribution values of the four biodiversity metrics from 48,870 cell-taxon combinations and maps of biogeographical sectors when using two biogeographical sectors (sensitivity analyses). Dots denote the median; thick and thin lines indicate the 66% and 95% quantile intervals, respectively.

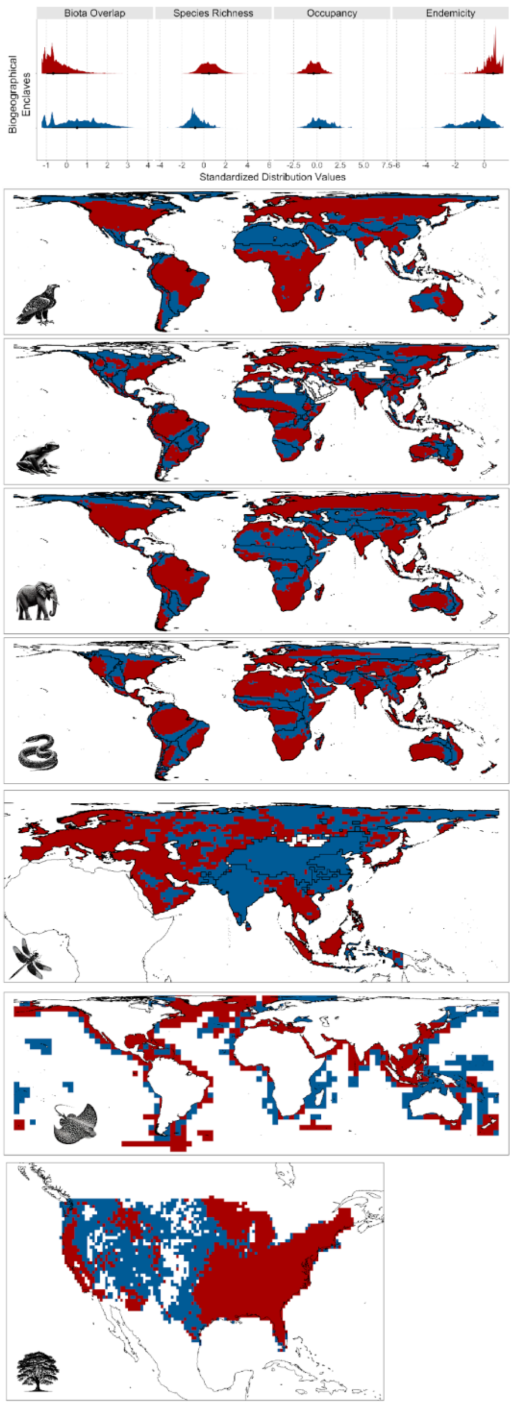

Supplementary Fig. 9 | Distribution values of the four biodiversity metrics and maps of biogeographical sectors when using three biogeographical sectors (sensitivity analyses). See details on sample size, dots and lines in Supplementary Fig. 8.

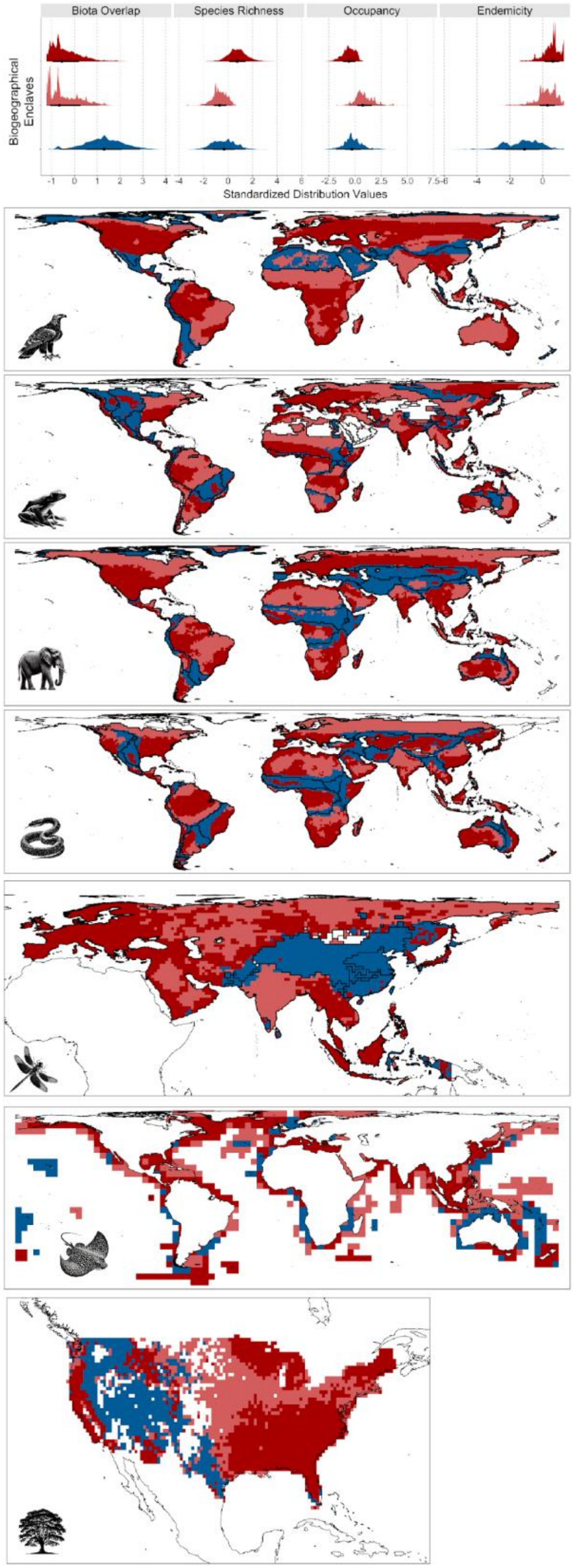

Supplementary Fig. 10 | Distribution values of the four biodiversity metrics and maps of biogeographical sectors when using four biogeographical sectors (sensitivity analyses). See details on sample size, dots and lines in Supplementary Fig. 8.

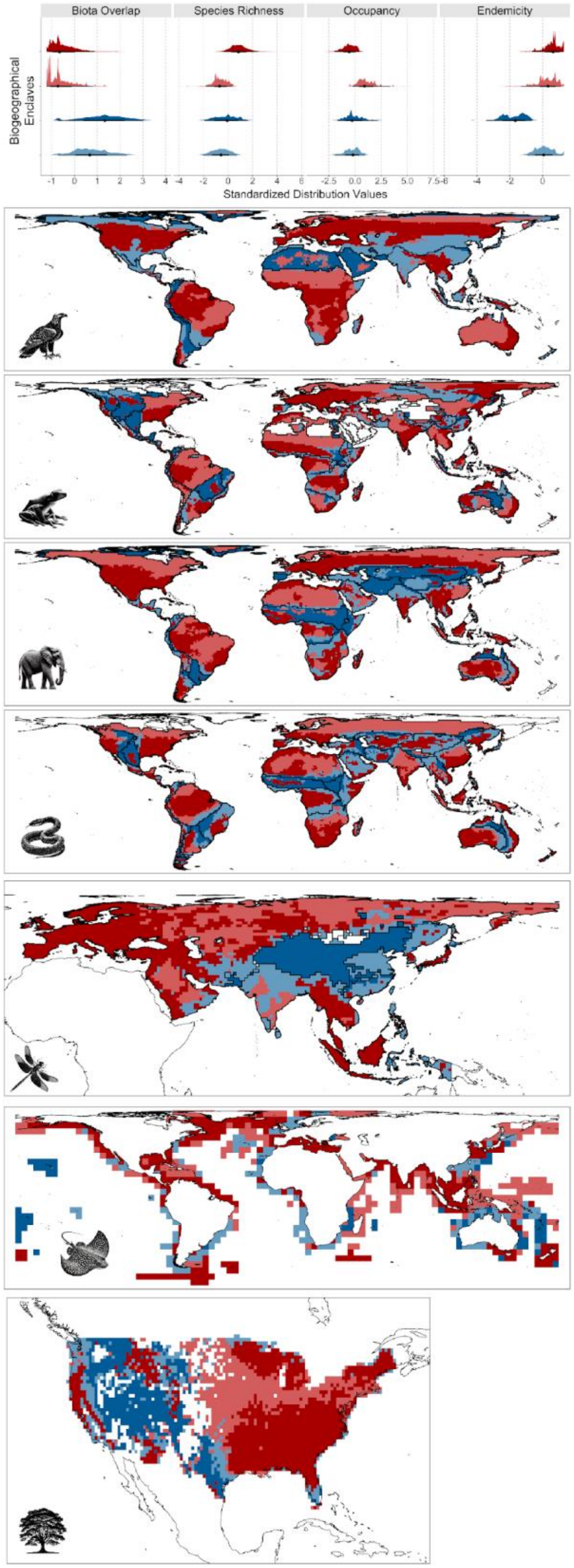

Supplementary Fig. 11 | Distribution values of the four biodiversity metrics and maps of biogeographical sectors when using five biogeographical sectors (sensitivity analyses). See details on sample size, dots and lines in Supplementary Fig. 8.

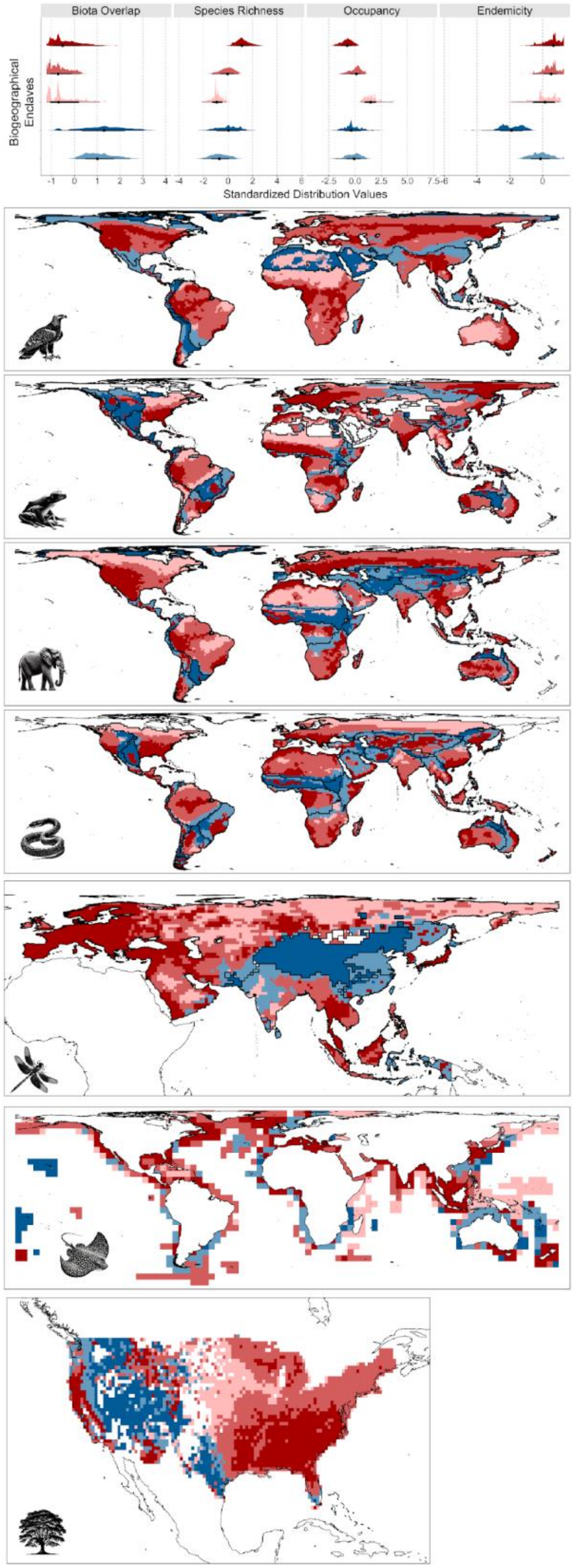

Supplementary Fig. 12 | Distribution values of the four biodiversity metrics and maps of biogeographical sectors when using six biogeographical sectors (sensitivity analyses). See details on sample size, dots and lines in Supplementary Fig. 8.

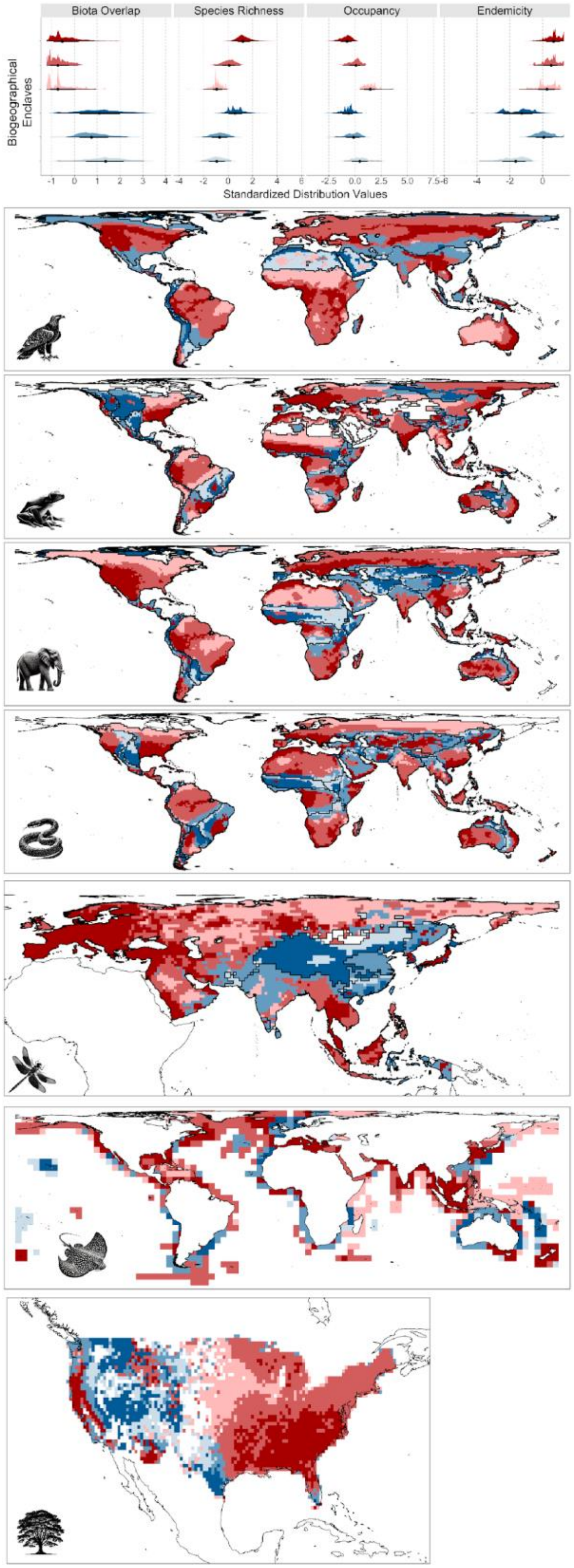

Supplementary Fig. 13 | Distribution values of the four biodiversity metrics and maps of biogeographical sectors when using eight biogeographical sectors (sensitivity analyses). See details on sample size, dots and lines in Supplementary Fig. 8.

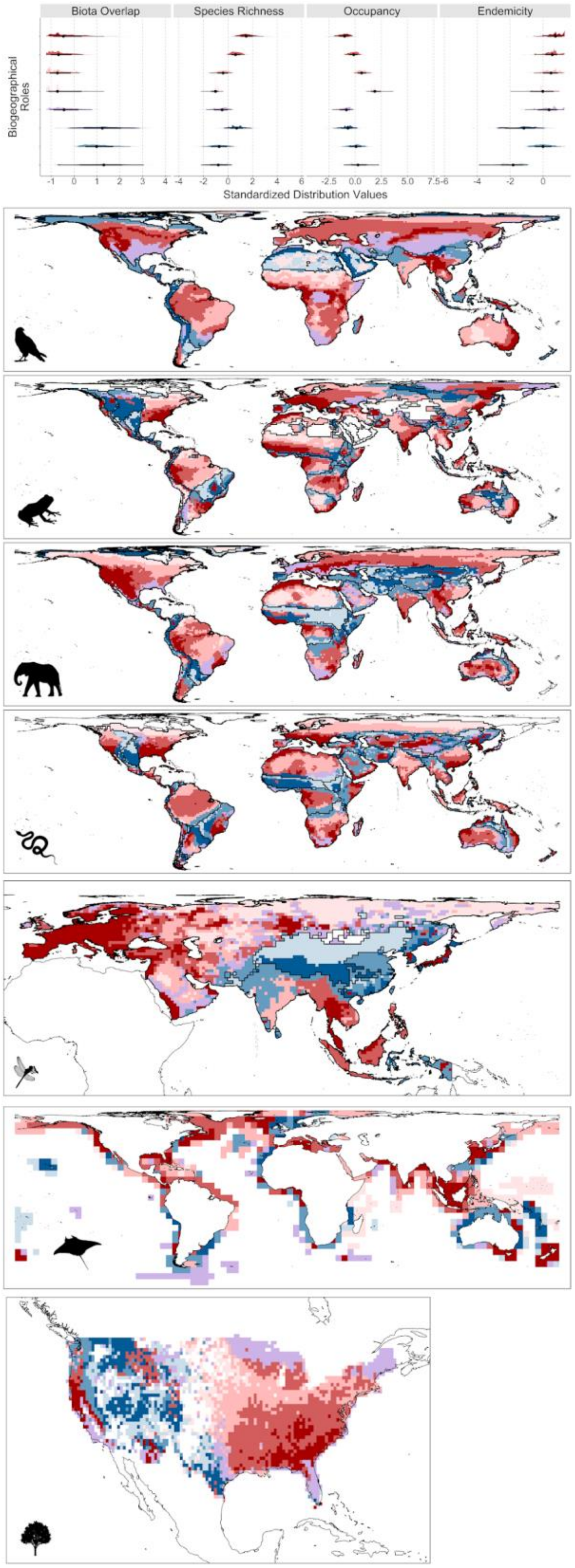

Supplementary Fig. 14 | Upper plot shows the apparent relative importance of precipitation and temperature in explaining differences between biogeographical sectors in each amphibian biogeographical region. We note that the relative importance of the variables can vary depending on the data and scale used as well as with the addition of other factors[80]. We therefore term it as apparent relative importance. Yellow and blue colours respectively indicate whether temperature or precipitation explain better the biogeographical sectors. Lower plot shows the McFadden's pseudo-R^2 of multinomial models of each bioregion. Grey colours indicate the proportion of the variance explained by the environmental variables.

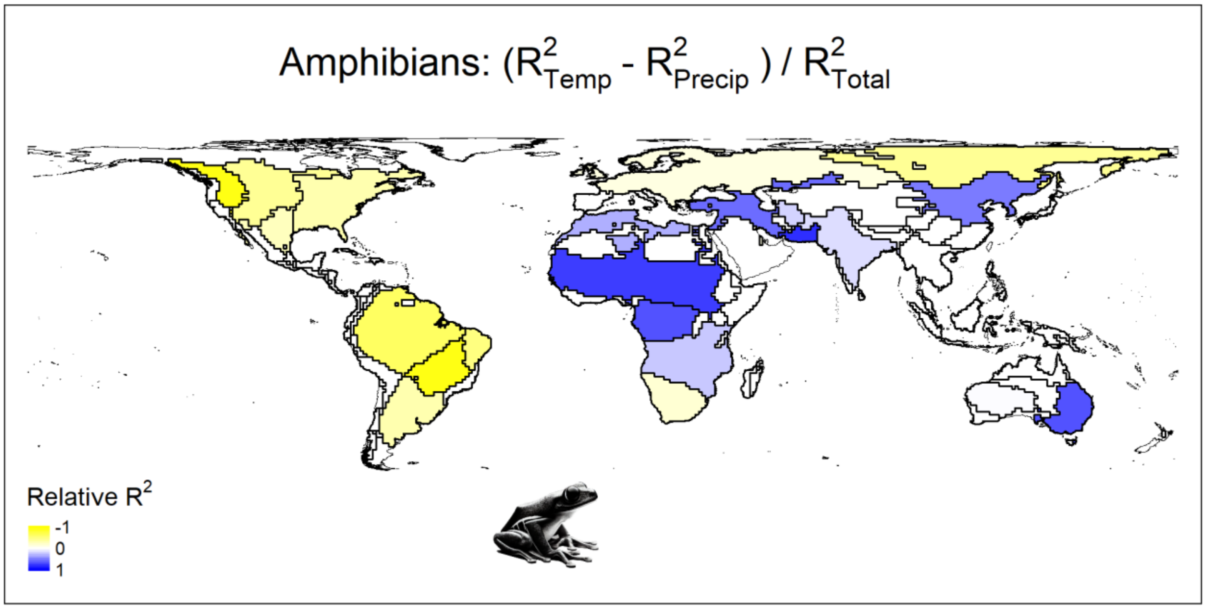

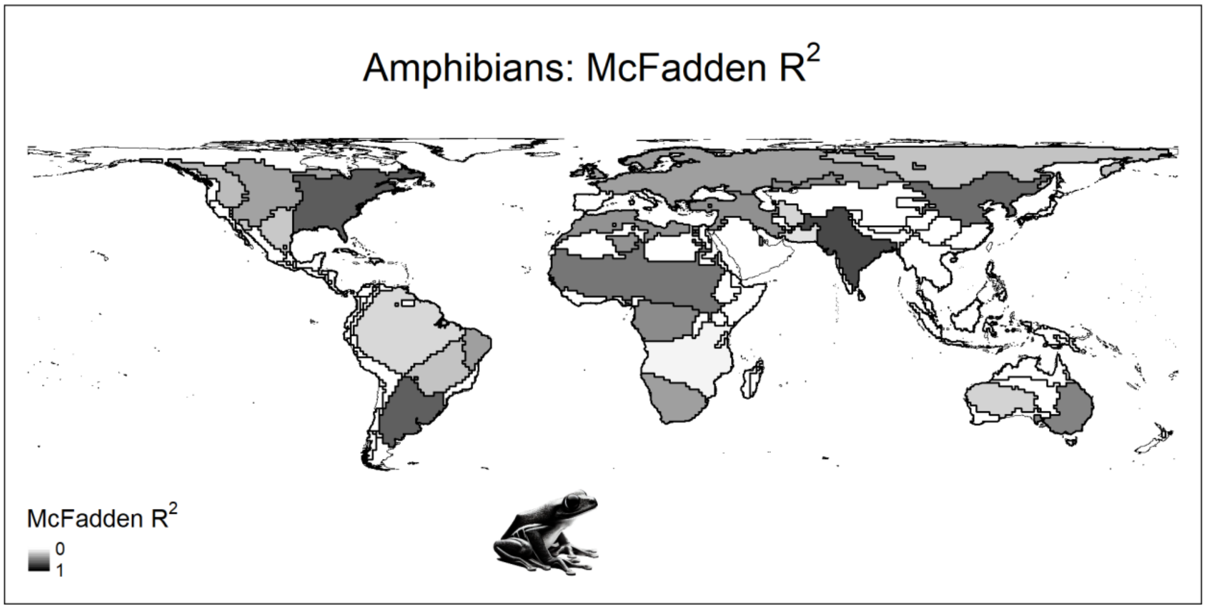

Supplementary Fig. 15 | Upper plot shows the apparent relative importance of precipitation and temperature in explaining differences between biogeographical sectors in each bird biogeographical region. Yellow and blue colours respectively indicate whether temperature or precipitation explain better the biogeographical sectors. Lower plot shows the McFadden's pseudo-R^2 of multinomial models of each bioregion. Grey colours indicate the proportion of the variance explained by the environmental variables. See comment about the "apparent" relative importance of the variables in caption of Supplementary Fig. 14.

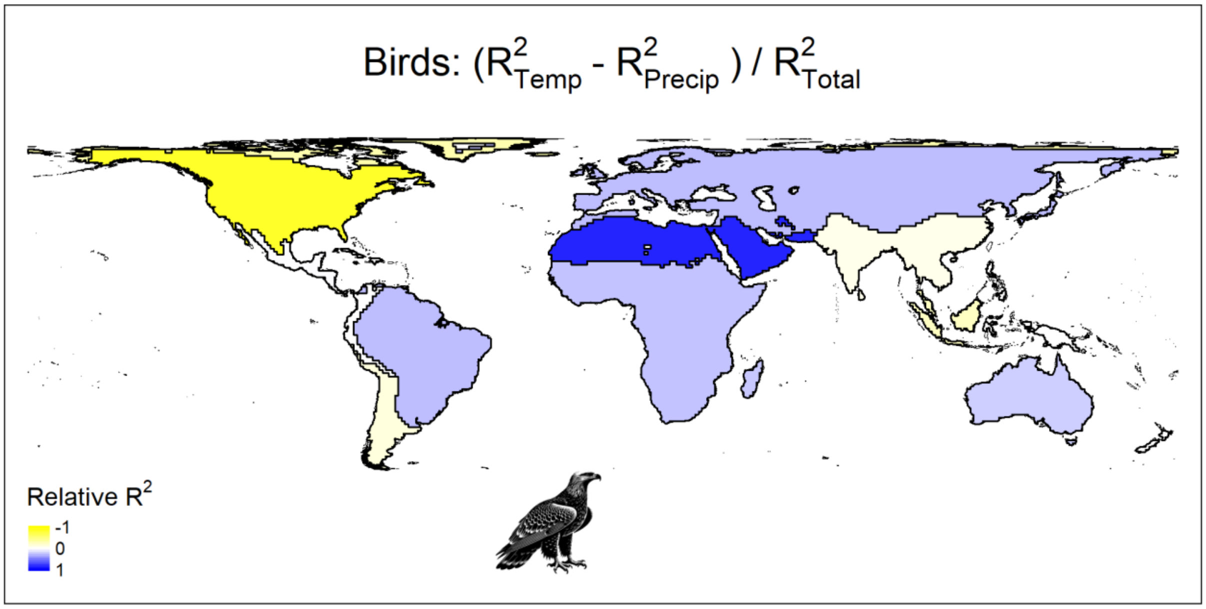

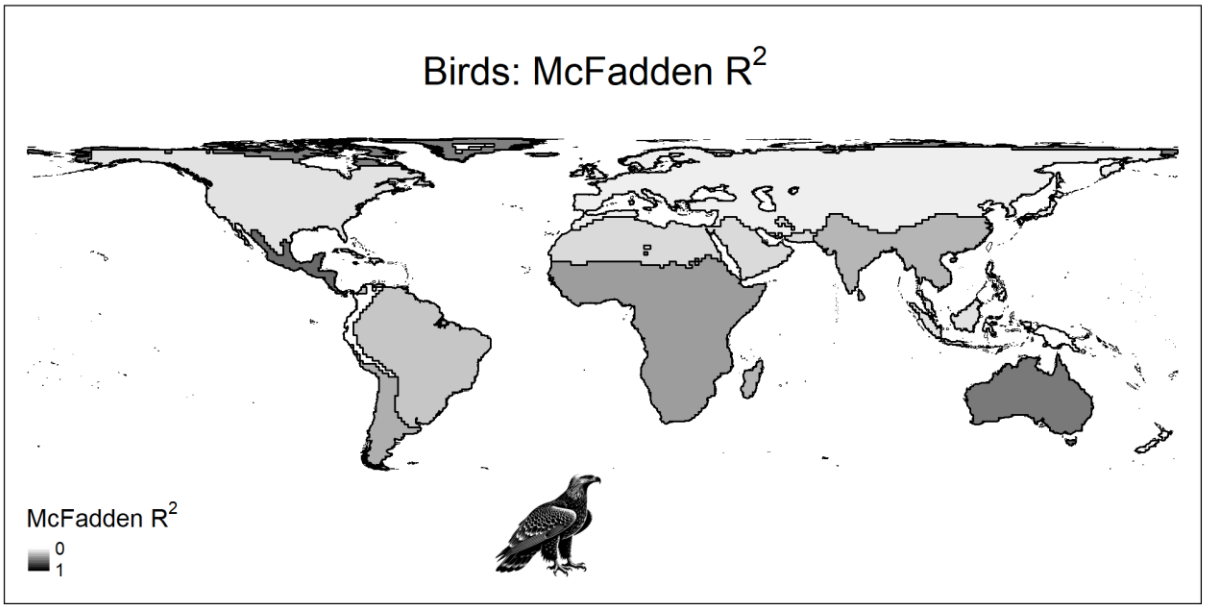

Supplementary Fig. 16 | Upper plot shows the apparent relative importance of precipitation and temperature in explaining differences between biogeographical sectors in each dragonfly biogeographical region. Yellow and blue colours respectively indicate whether temperature or precipitation explain better the biogeographical sectors. Lower plot shows the McFadden's pseudo-R^2 of multinomial models of each bioregion. Grey colours indicate the proportion of the variance explained by the environmental variables. See comment about the "apparent" relative importance of the variables in caption of Supplementary Fig. 14.

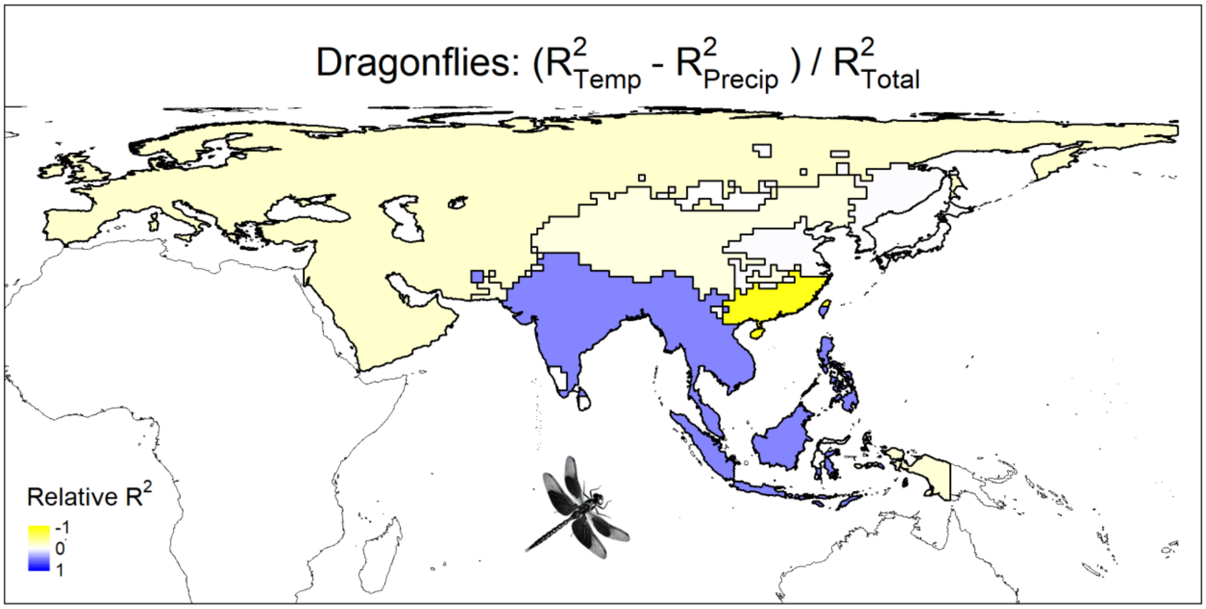

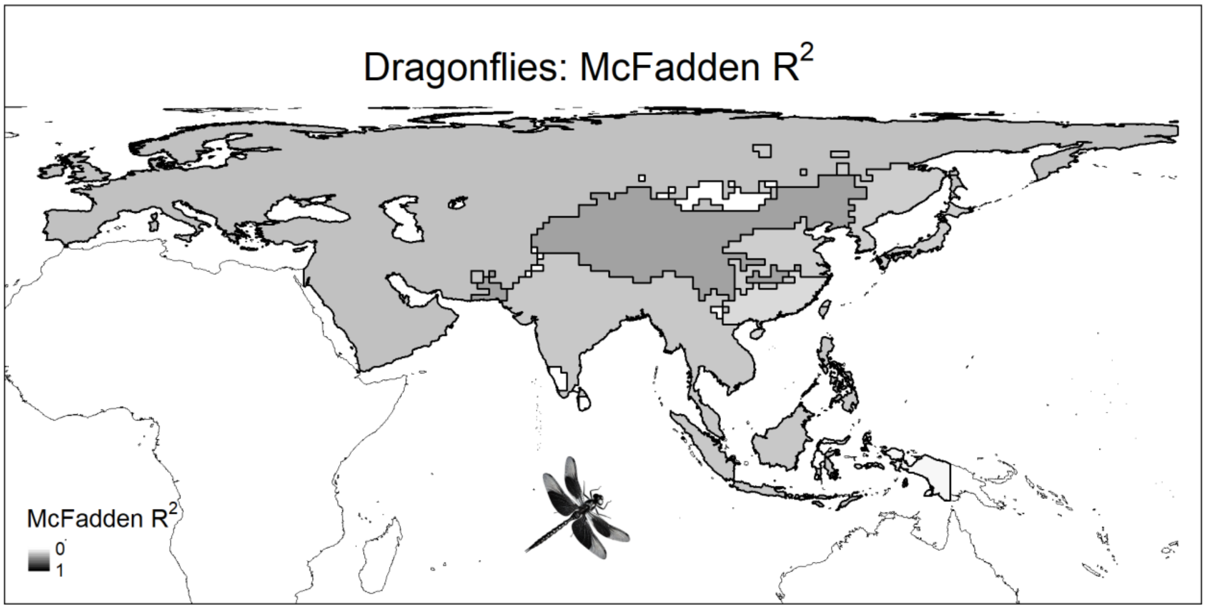

Supplementary Fig. 17 | Upper plot shows the apparent relative importance of precipitation and temperature in explaining differences between biogeographical sectors in each mammal biogeographical region. Yellow and blue colours respectively indicate whether temperature or precipitation explain better the biogeographical sectors. Lower plot shows the McFadden's pseudo-R^2 of multinomial models of each bioregion. Grey colours indicate the proportion of the variance explained by the environmental variables. See comment about the "apparent" relative importance of the variables in caption of Supplementary Fig. 14.

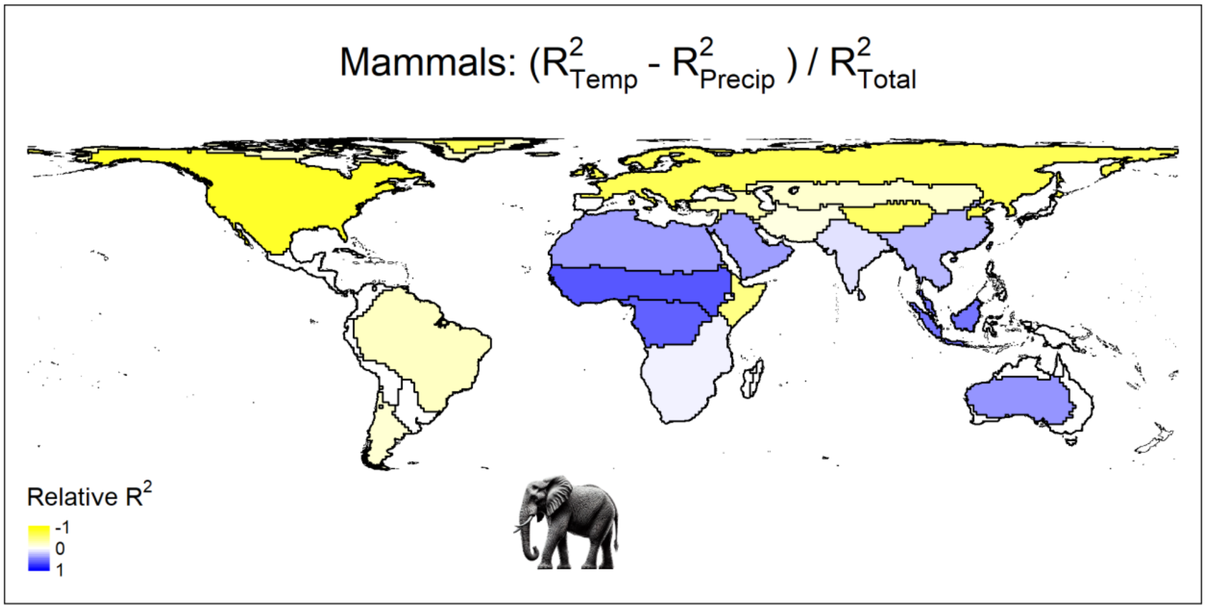

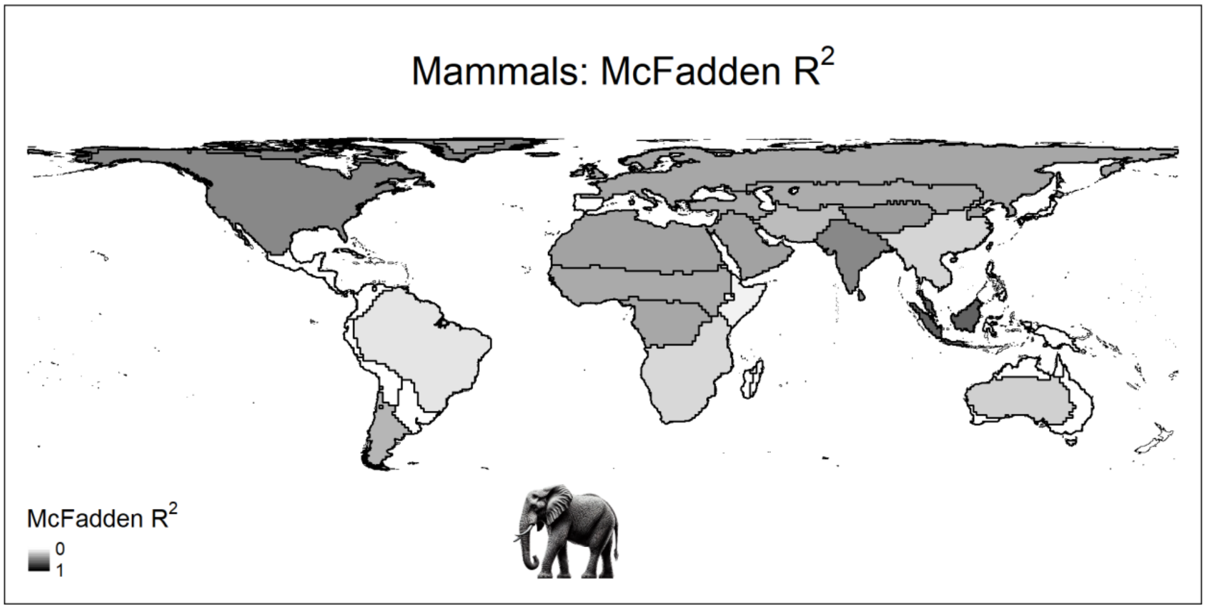

Supplementary Fig. 18 | Upper plot shows the apparent relative importance of precipitation and temperature in explaining differences between biogeographical sectors in each ray biogeographical region. Yellow and blue colours respectively indicate whether temperature or precipitation explain better the biogeographical sectors. Lower plot shows the McFadden's pseudo-R^2 of multinomial models of each bioregion. Grey colours indicate the proportion of the variance explained by the environmental variables. See comment about the "apparent" relative importance of the variables in caption of Supplementary Fig. 14.

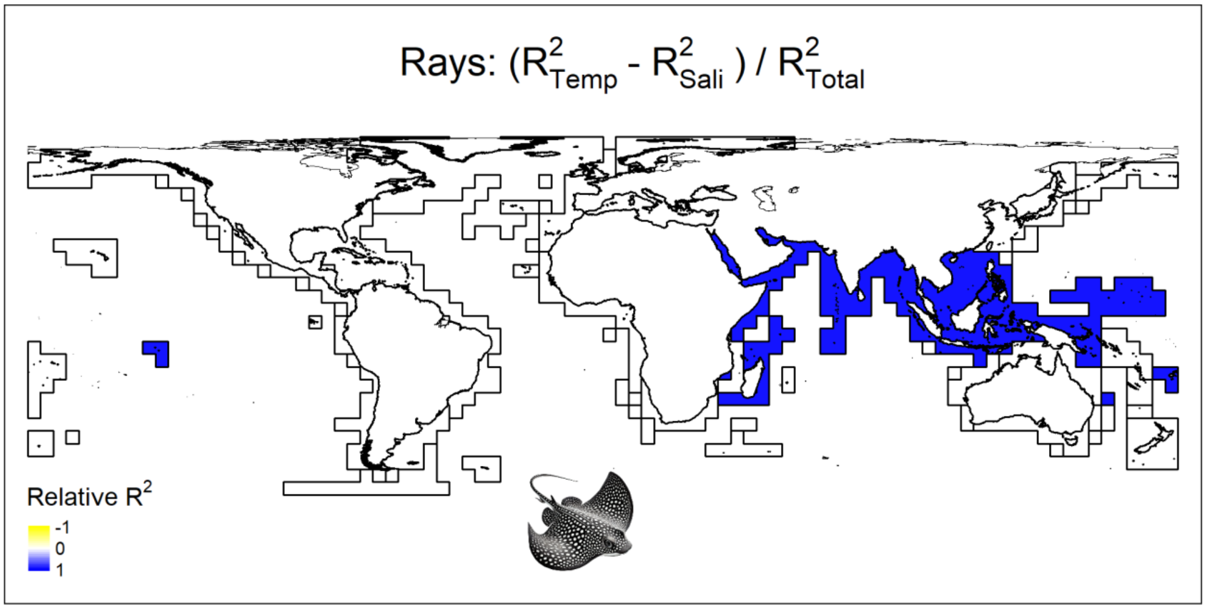

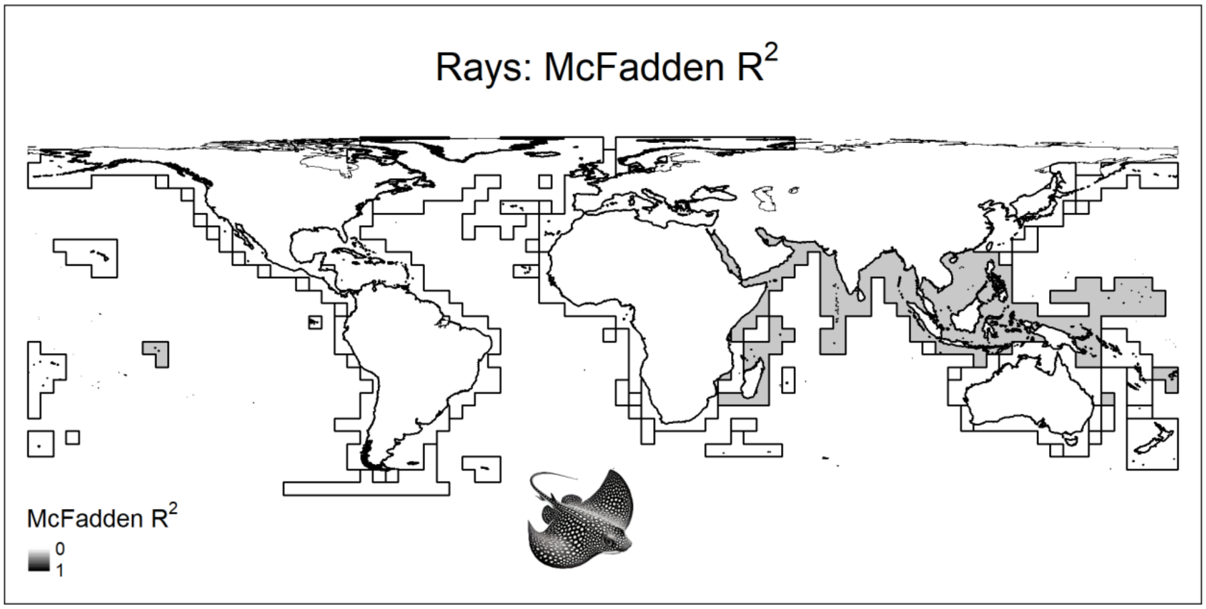

Supplementary Fig. 19 | Upper plot shows the apparent relative importance of precipitation and temperature in explaining differences between biogeographical sectors in each reptile biogeographical region. Yellow and blue colours respectively indicate whether temperature or precipitation explain better the biogeographical sectors. Lower plot shows the McFadden's pseudo-R^2 of multinomial models of each bioregion. Grey colours indicate the proportion of the variance explained by the environmental variables. See comment about the "apparent" relative importance of the variables in caption of Supplementary Fig. 14.

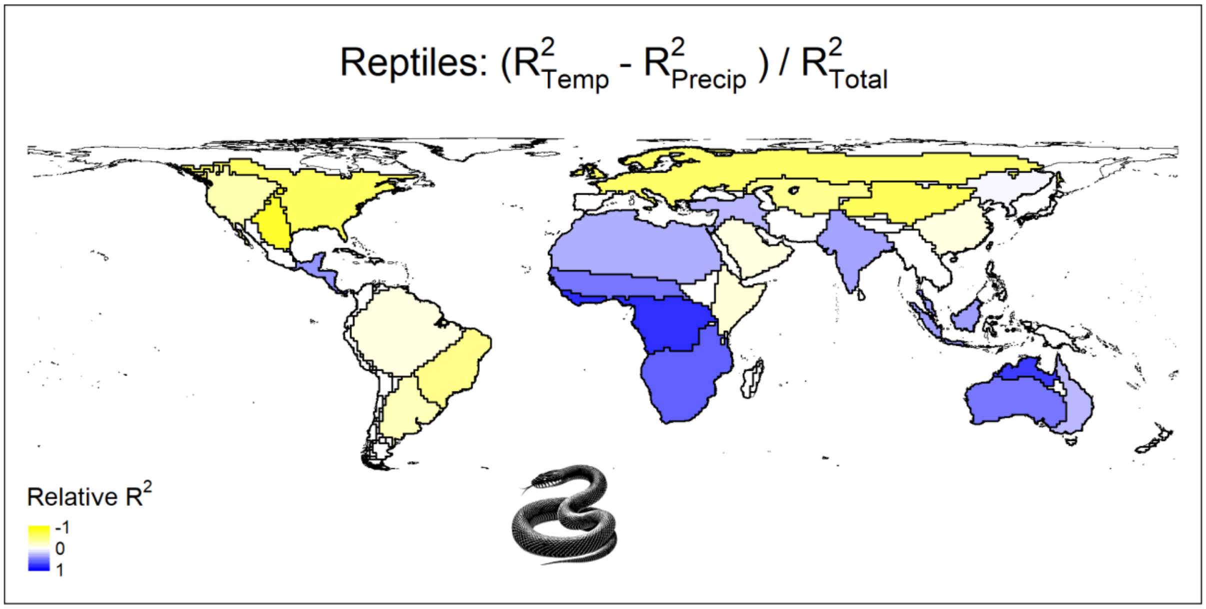

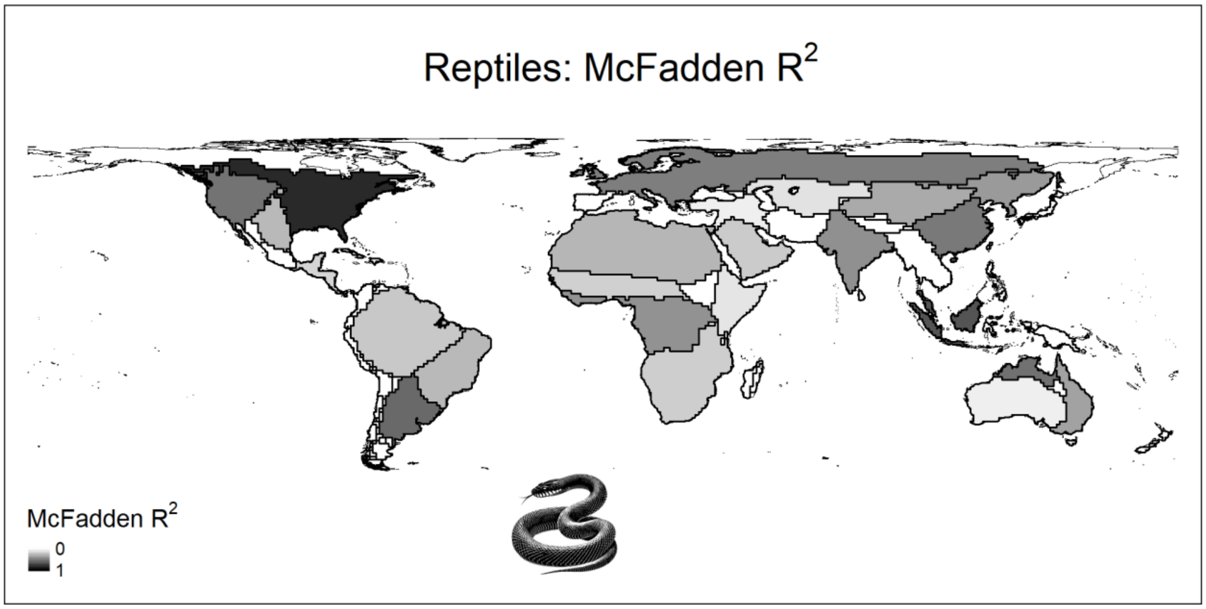

Supplementary Fig. 20 | Upper plot shows the apparent relative importance of precipitation and temperature in explaining differences between biogeographical sectors in each tree biogeographical region. Yellow and blue colours respectively indicate whether temperature or precipitation explain better the biogeographical sectors. Lower plot shows the McFadden's pseudo-R^2 of multinomial models of each bioregion. Grey colours indicate the proportion of the variance explained by the environmental variables. See comment about the "apparent" relative importance of the variables in caption of Supplementary Fig. 14.

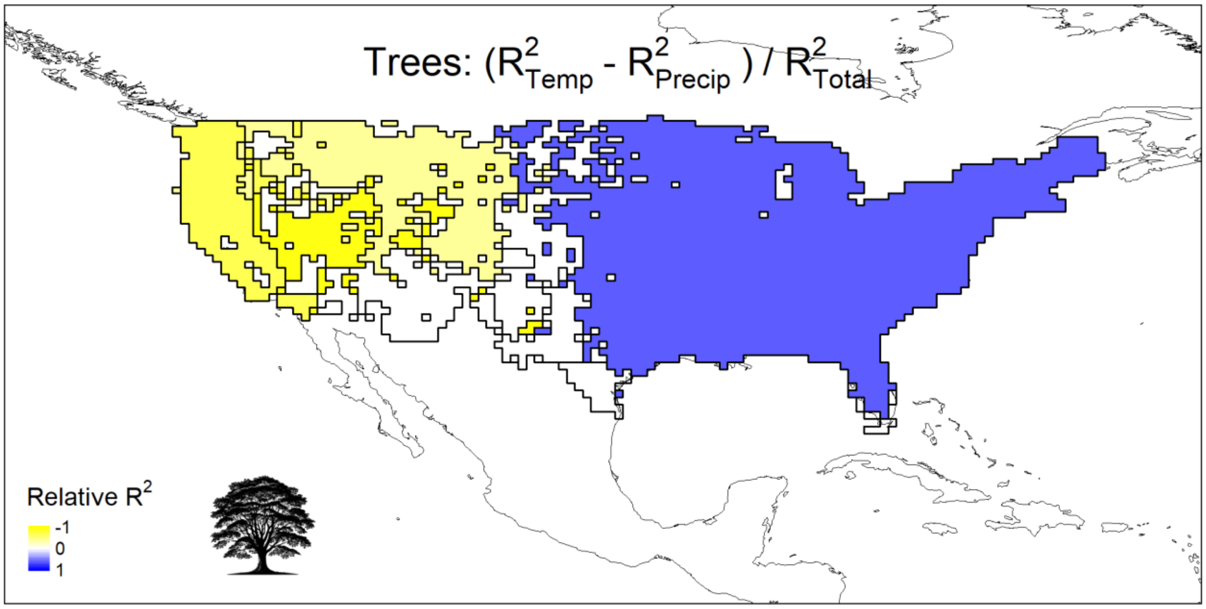

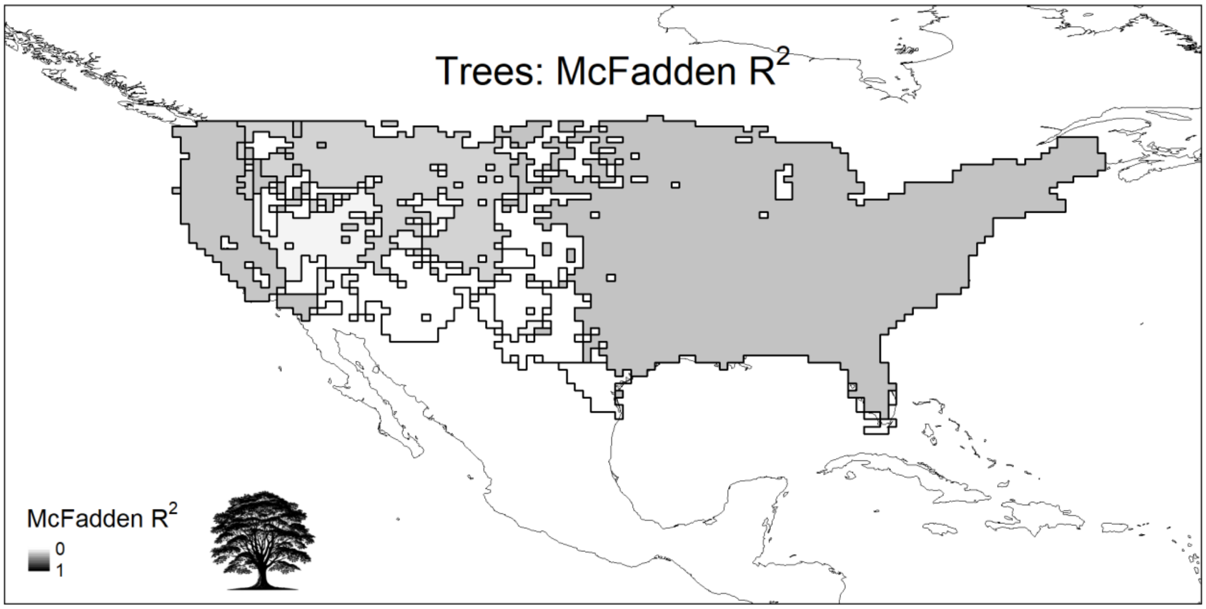

Supplementary Fig. 21 | The upper plot shows the apparent relative importance of present climate conditions versus the climate change since the Last Glacial Maximum in explaining biogeographical sectors across each amphibian biogeographical region. Blue and brown colours respectively indicate whether present climate or climate change since the LGM serves as a better predictor of biogeographical sectors. The lower plot shows the McFadden's pseudo-R^2 of multinomial models of each bioregion. Grey colours indicate the proportion of the variance explained by the environmental variables. See comment about the "apparent" relative importance of the variables in caption of Supplementary Fig. 14.

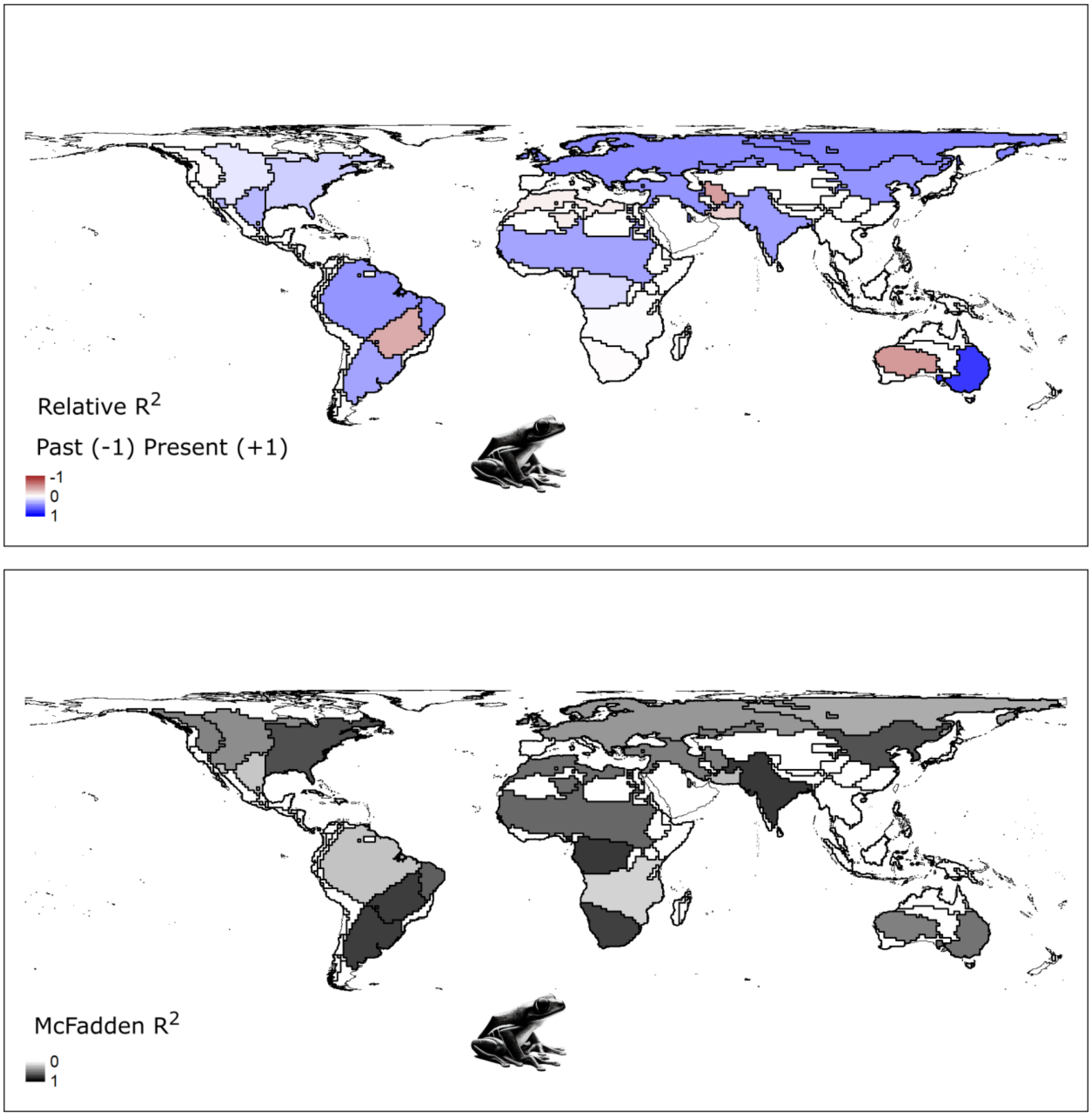

Supplementary Fig. 22 | The upper plot shows the apparent relative importance of present climate conditions versus the climate change since the Last Glacial Maximum in explaining biogeographical sectors across each bird biogeographical region. Blue and brown colours respectively indicate whether present climate or climate change since the LGM serves as a better predictor of biogeographical sectors. The lower plot shows the McFadden's pseudo-R^2 of multinomial models of each bioregion. Grey colours indicate the proportion of the variance explained by the environmental variables. See comment about the "apparent" relative importance of the variables in caption of Supplementary Fig. 14.

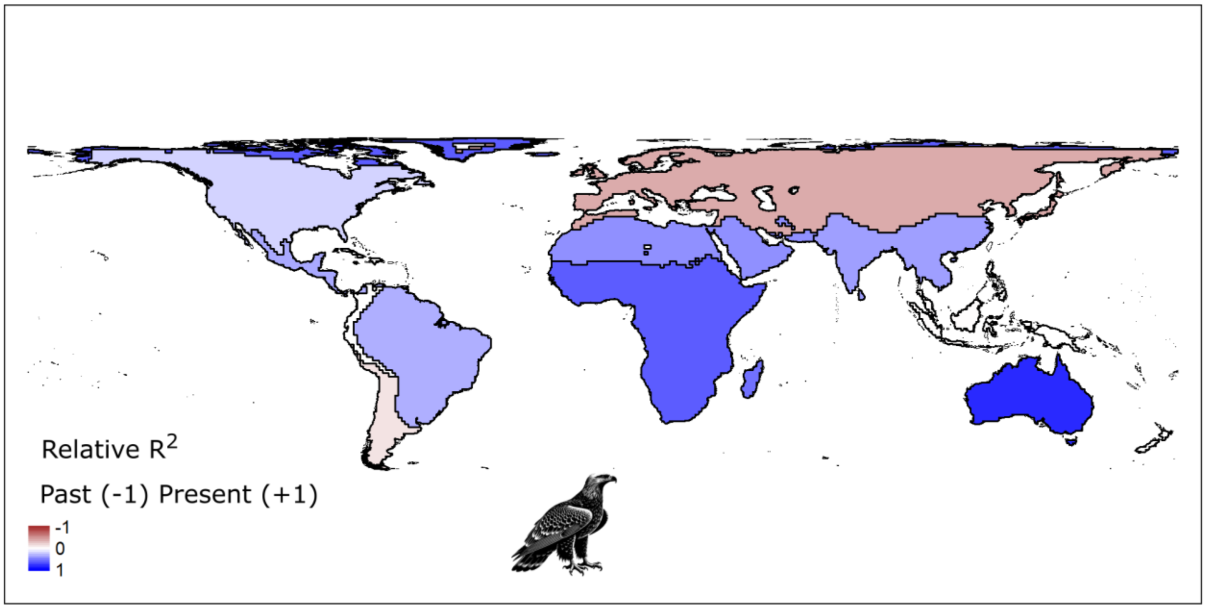

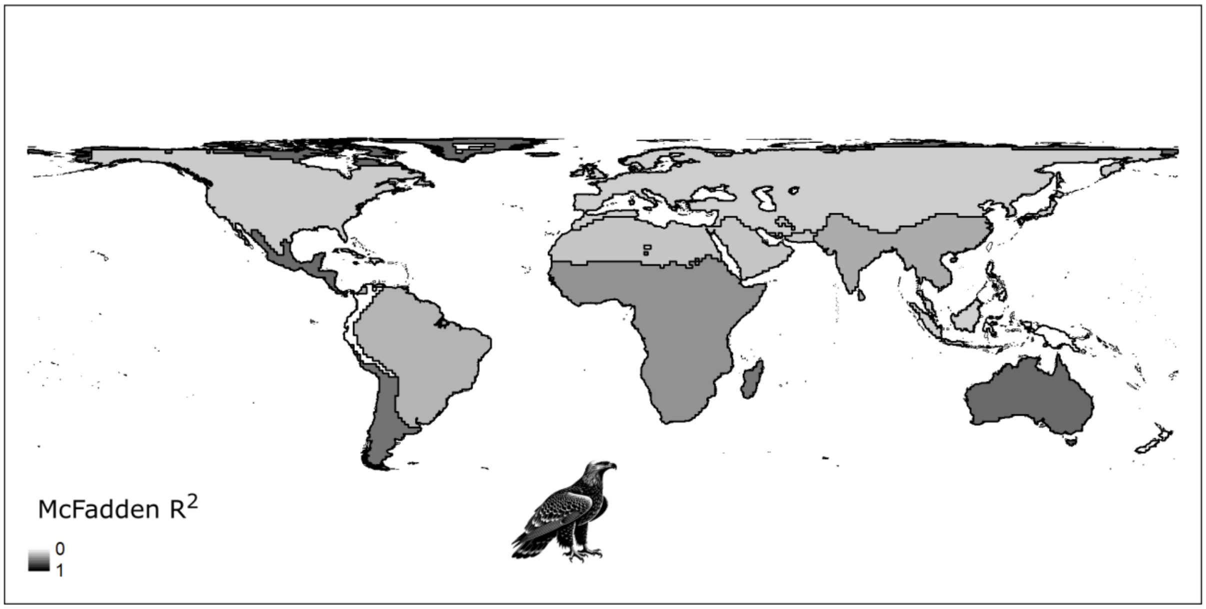

Supplementary Fig. 23 | The upper plot shows the apparent relative importance of present climate conditions versus the climate change since the Last Glacial Maximum in explaining biogeographical sectors across each dragonfly biogeographical region. Blue and brown colours respectively indicate whether present climate or climate change since the LGM serves as a better predictor of biogeographical sectors. The lower plot shows the McFadden's pseudo-R^2 of multinomial models of each bioregion. Grey colours indicate the proportion of the variance explained by the environmental variables. See comment about the "apparent" relative importance of the variables in caption of Supplementary Fig. 14.

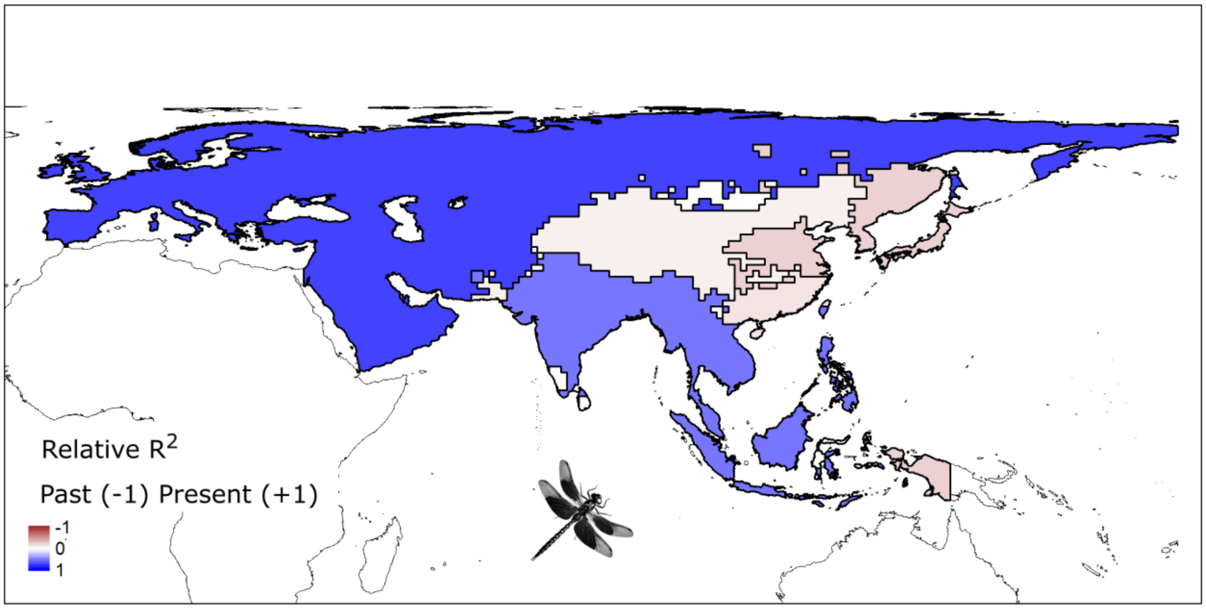

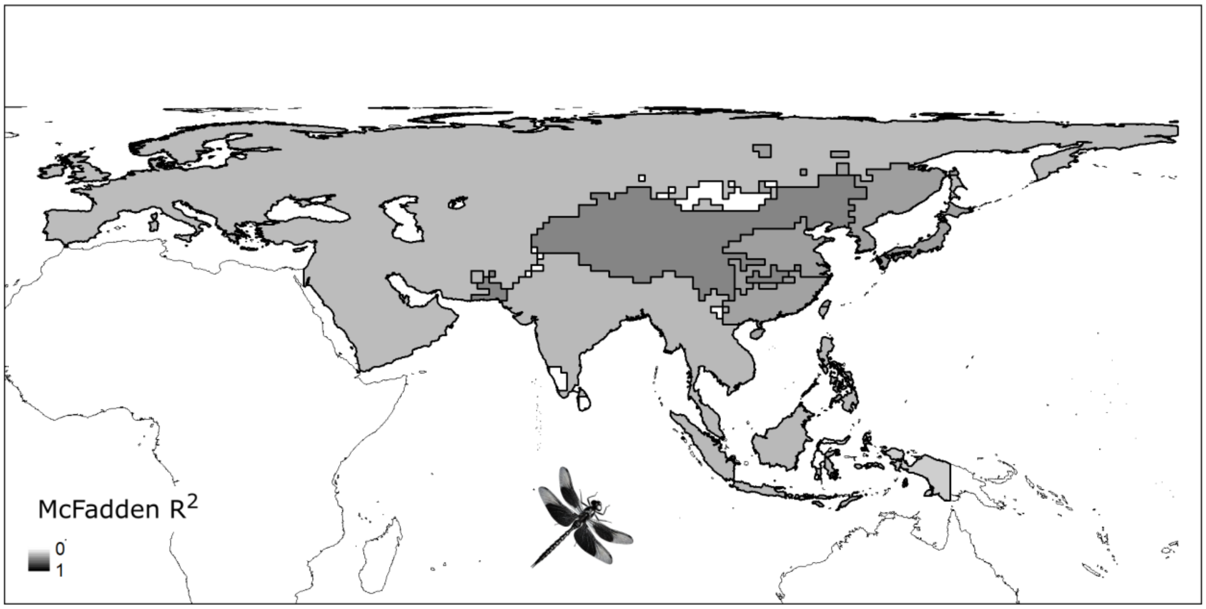

Supplementary Fig. 24 | The upper plot shows the apparent relative importance of present climate conditions versus the climate change since the Last Glacial Maximum in explaining biogeographical sectors across each mammal biogeographical region. Blue and brown colours respectively indicate whether present climate or climate change since the LGM serves as a better predictor of biogeographical sectors. The lower plot shows the McFadden's pseudo-R^2 of multinomial models of each bioregion. Grey colours indicate the proportion of the variance explained by the environmental variables. See comment about the "apparent" relative importance of the variables in caption of Supplementary Fig. 14.

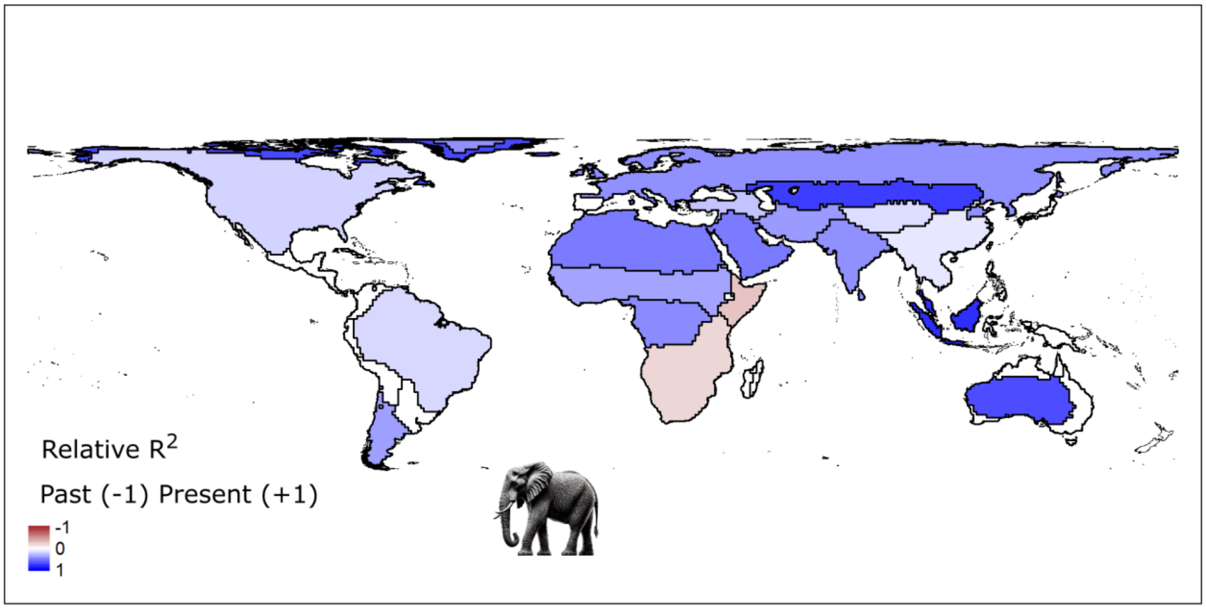

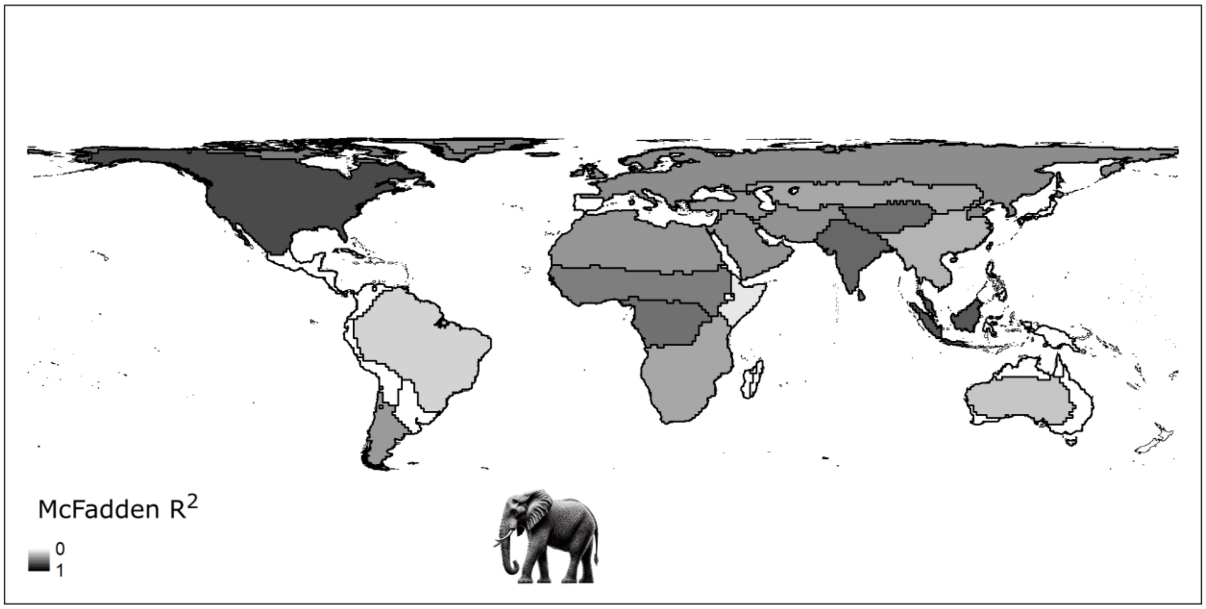

Supplementary Fig. 25 | The upper plot shows the apparent relative importance of present climate conditions versus the climate change since the Last Glacial Maximum in explaining biogeographical sectors across each reptile biogeographical region. Blue and brown colours respectively indicate whether present climate or climate change since the LGM serves as a better predictor of biogeographical sectors. The lower plot shows the McFadden's pseudo-R^2 of multinomial models of each bioregion. Grey colours indicate the proportion of the variance explained by the environmental variables. See comment about the "apparent" relative importance of the variables in caption of Supplementary Fig. 14.

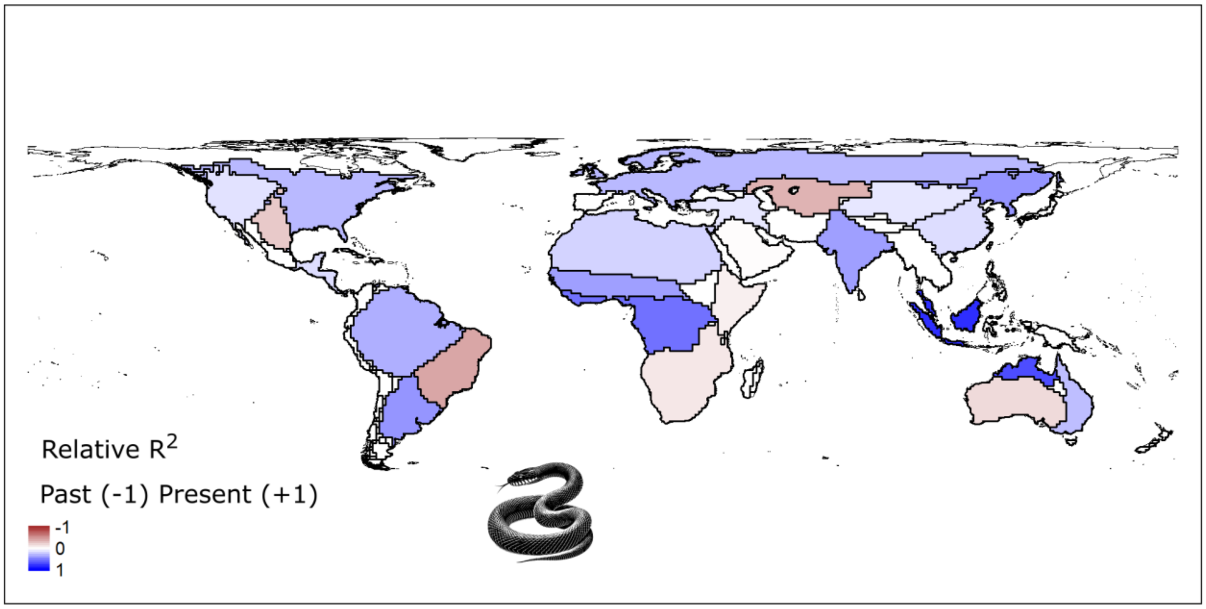

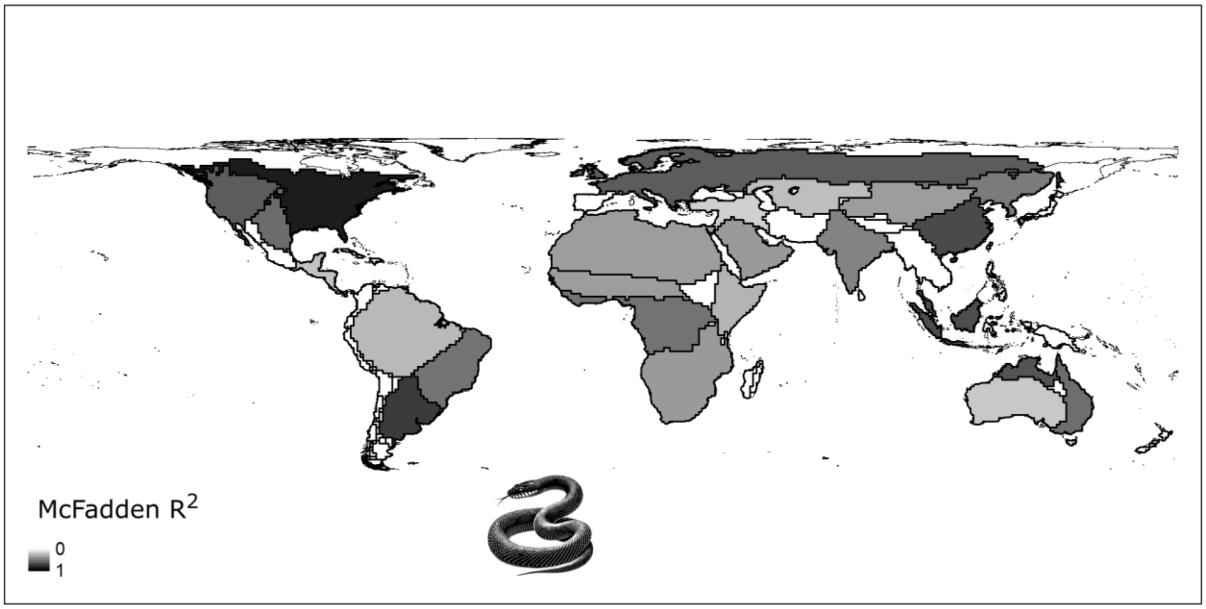

Supplementary Fig. 26 | The upper plot shows the apparent relative importance of present climate conditions versus the climate change since the Last Glacial Maximum in explaining biogeographical sectors across each tree biogeographical region. Blue and brown colours respectively indicate whether present climate or climate change since the LGM serves as a better predictor of biogeographical sectors. The lower plot shows the McFadden's pseudo-R^2 of multinomial models of each bioregion. Grey colours indicate the proportion of the variance explained by the environmental variables. See comment about the "apparent" relative importance of the variables in caption of Supplementary Fig. 14.

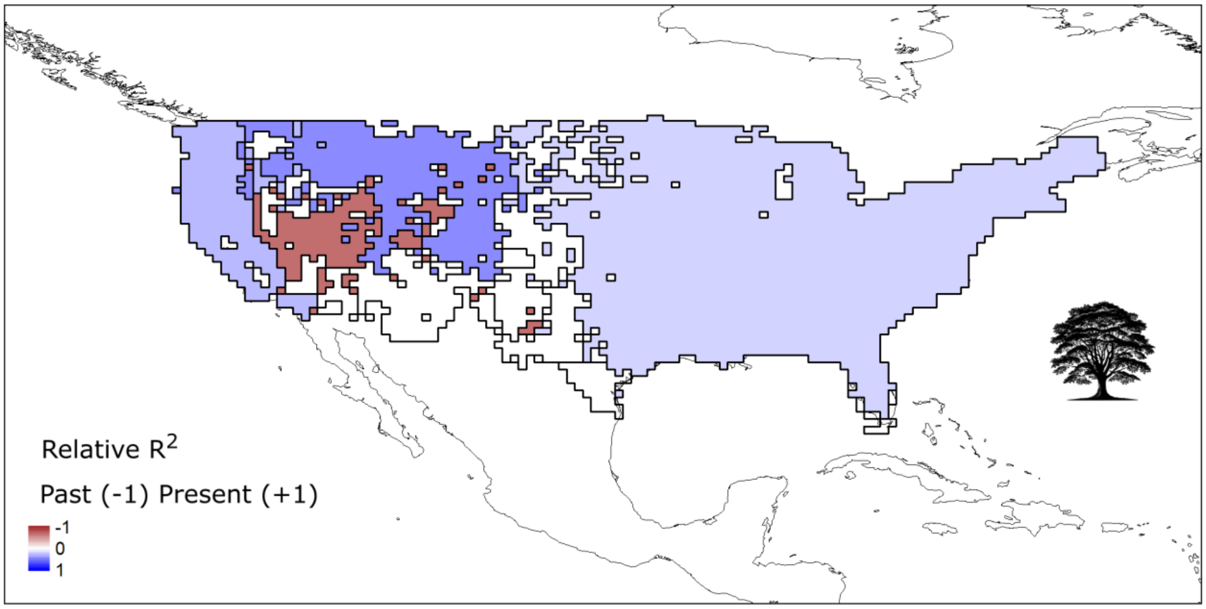

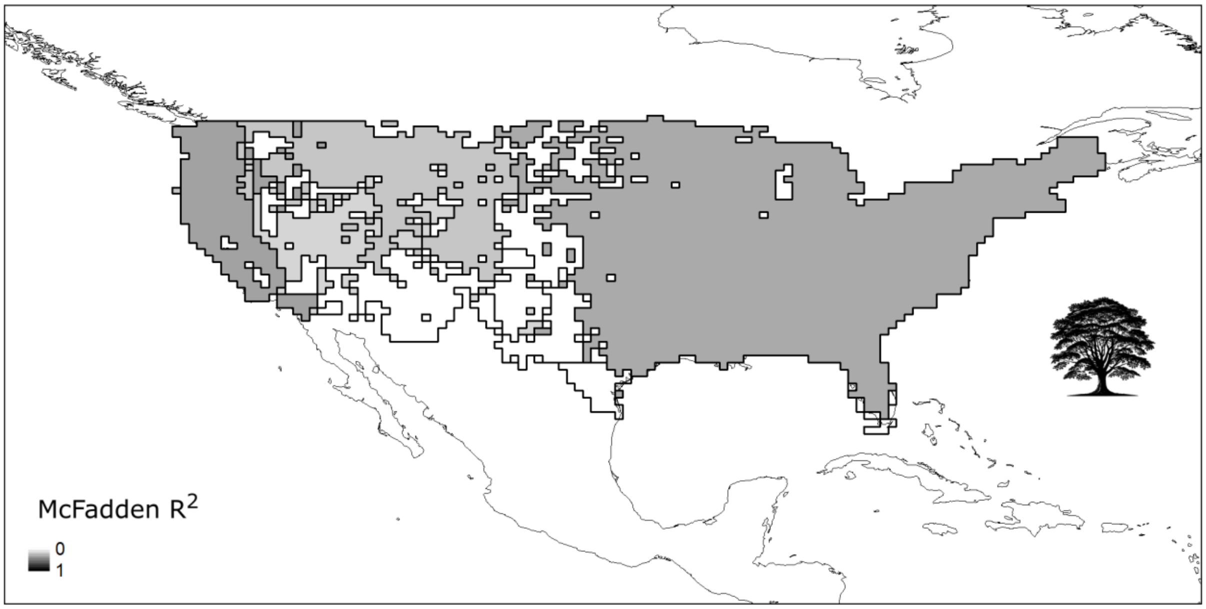

Supplementary Fig. 27 | Distribution of biodiversity values from 48,870 cell-taxon combinations across sectors represented by colours. Dots denote the median; thick and thin lines indicate the 66% and 95% quantile intervals, respectively. Biogeographical sectors when weighting the k-means by the number of observations per taxa. This kind of weighting provides more importance for the biogeographical sectors located in Eurasia and North America due to the lower extent of the study area in dragonflies and trees, respectively.

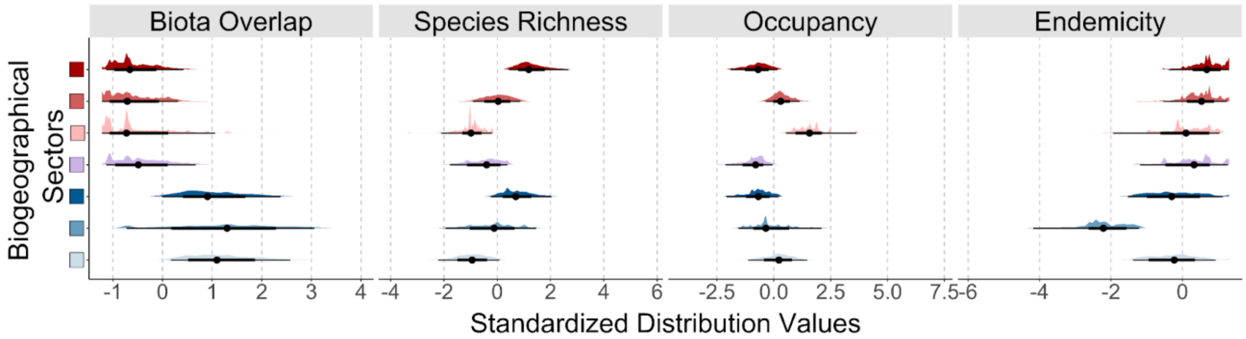

Supplementary Fig. 28 | Biogeographical sectors in amphibian biogeographical regions when weighting the k-means by the number of observations per taxa. This kind of weighting provides more importance for the biogeographical sectors located in Eurasia and North America due to the lower extent of the study area in dragonflies and trees, respectively.

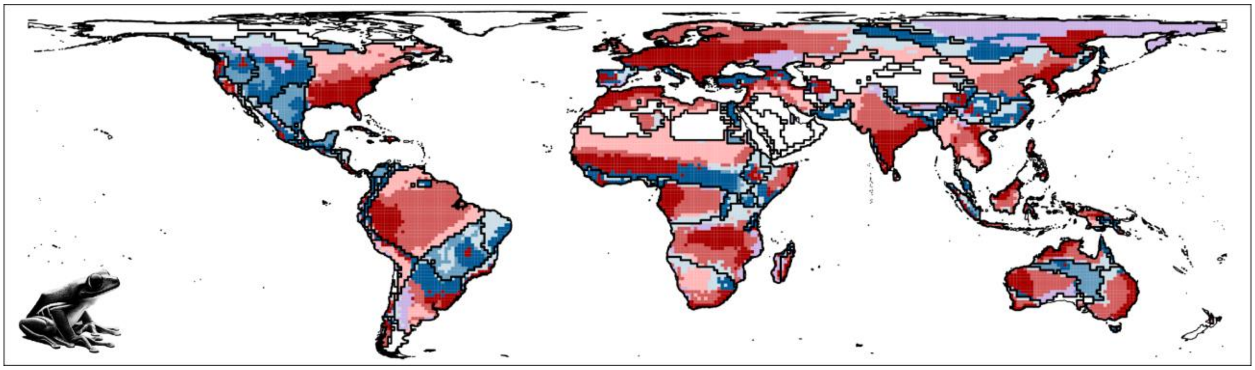

Supplementary Fig. 29 | Biogeographical sectors in bird biogeographical regions when weighting the k-means by the number of observations per taxa. This kind of weighting provides more importance for the biogeographical sectors located in Eurasia and North America due to the lower extent of the study area in dragonflies and trees, respectively.

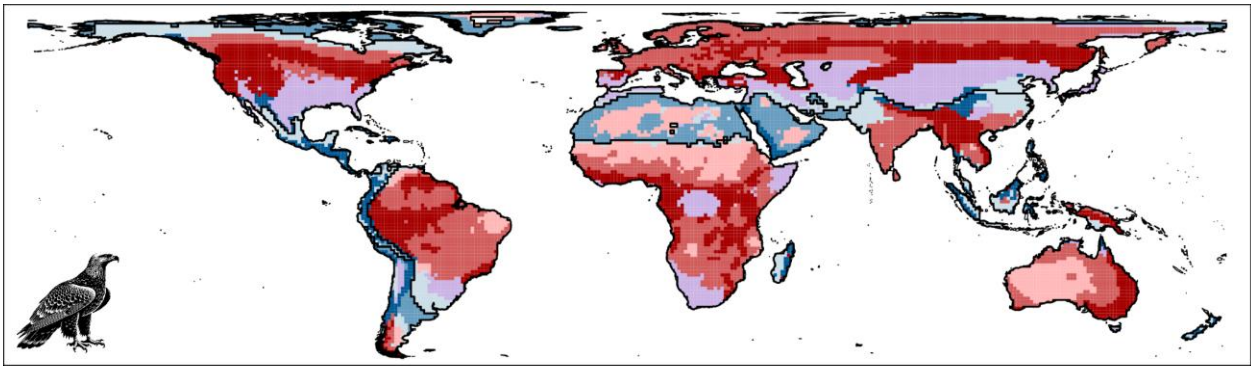

Supplementary Fig. 30 | Biogeographical sectors in mammal biogeographical regions when weighting the k-means by the number of observations per taxa. This kind of weighting provides more importance for the biogeographical sectors located in Eurasia and North America due to the lower extent of the study area in dragonflies and trees, respectively.

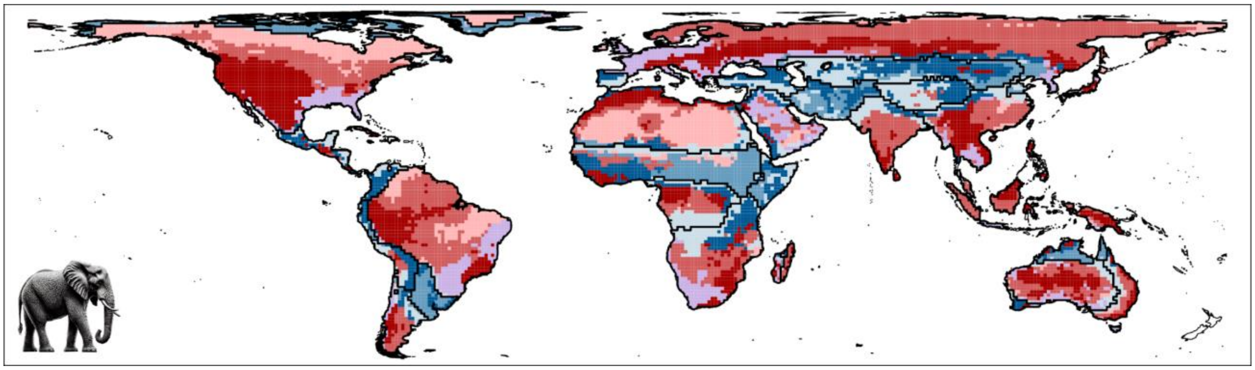

Supplementary Fig. 31 | Biogeographical sectors in dragonfly biogeographical regions when weighting the k-means by the number of observations per taxa. This kind of weighting provides more importance for the biogeographical sectors located in Eurasia and North America due to the lower extent of the study area in dragonflies and trees, respectively.

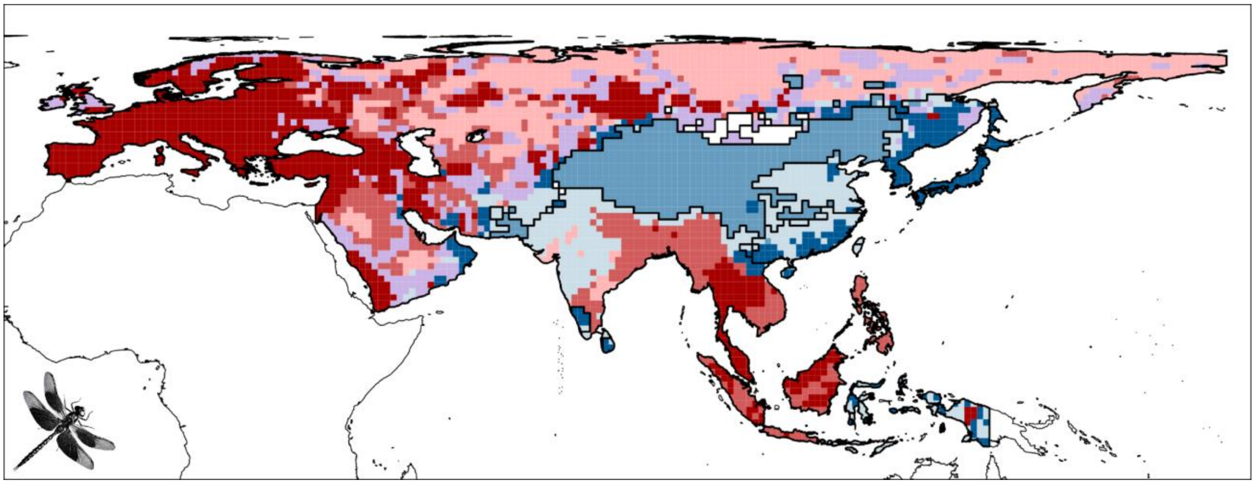

Supplementary Fig. 32 | Biogeographical sectors in ray biogeographical regions when weighting the k-means by the number of observations per taxa. This kind of weighting provides more importance for the biogeographical sectors located in Eurasia and North America due to the lower extent of the study area in dragonflies and trees, respectively.

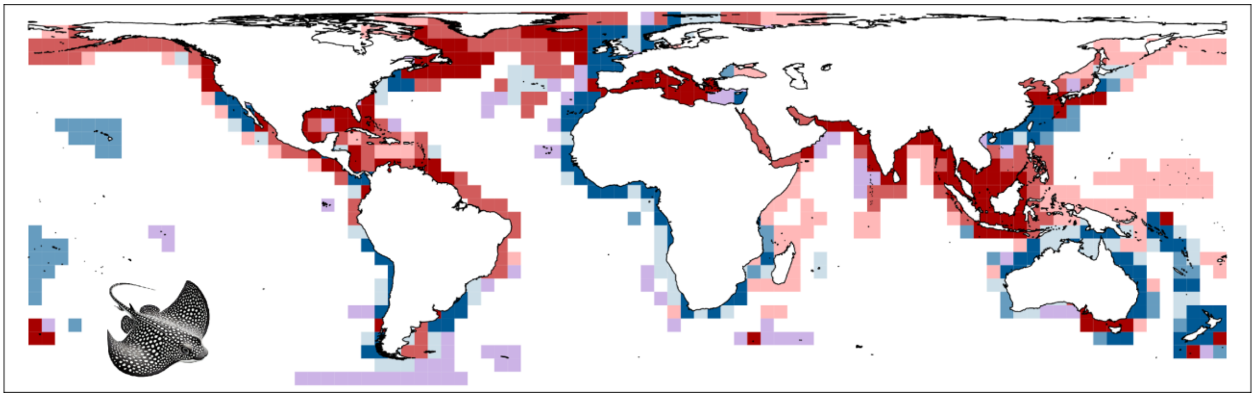

Supplementary Fig. 33 | Biogeographical sectors in reptile biogeographical regions when weighting the k-means by the number of observations per taxa. This kind of weighting provides more importance for the biogeographical sectors located in Eurasia and North America due to the lower extent of the study area in dragonflies and trees, respectively.

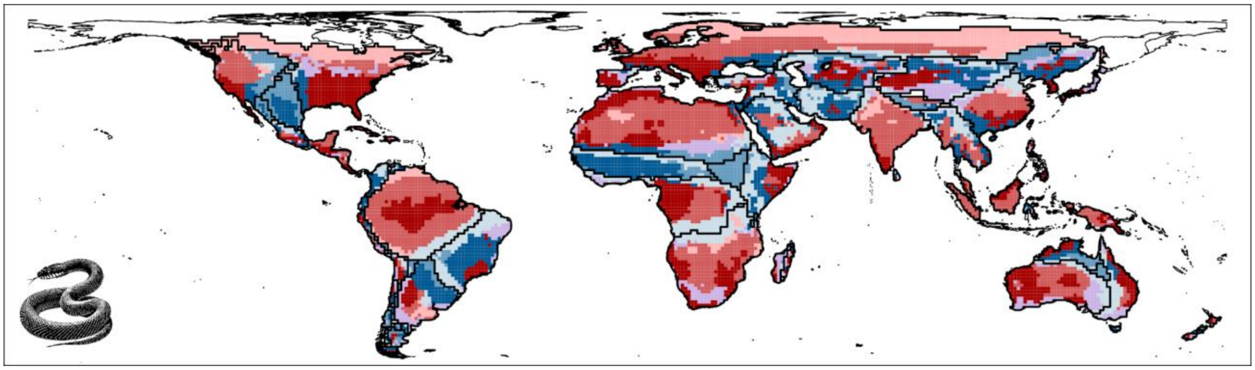

Supplementary Fig. 34 | Biogeographical sectors in tree biogeographical regions when weighting the k-means by the number of observations per taxa. This kind of weighting provides more importance for the biogeographical sectors located in Eurasia and North America due to the lower extent of the study area in dragonflies and trees, respectively.

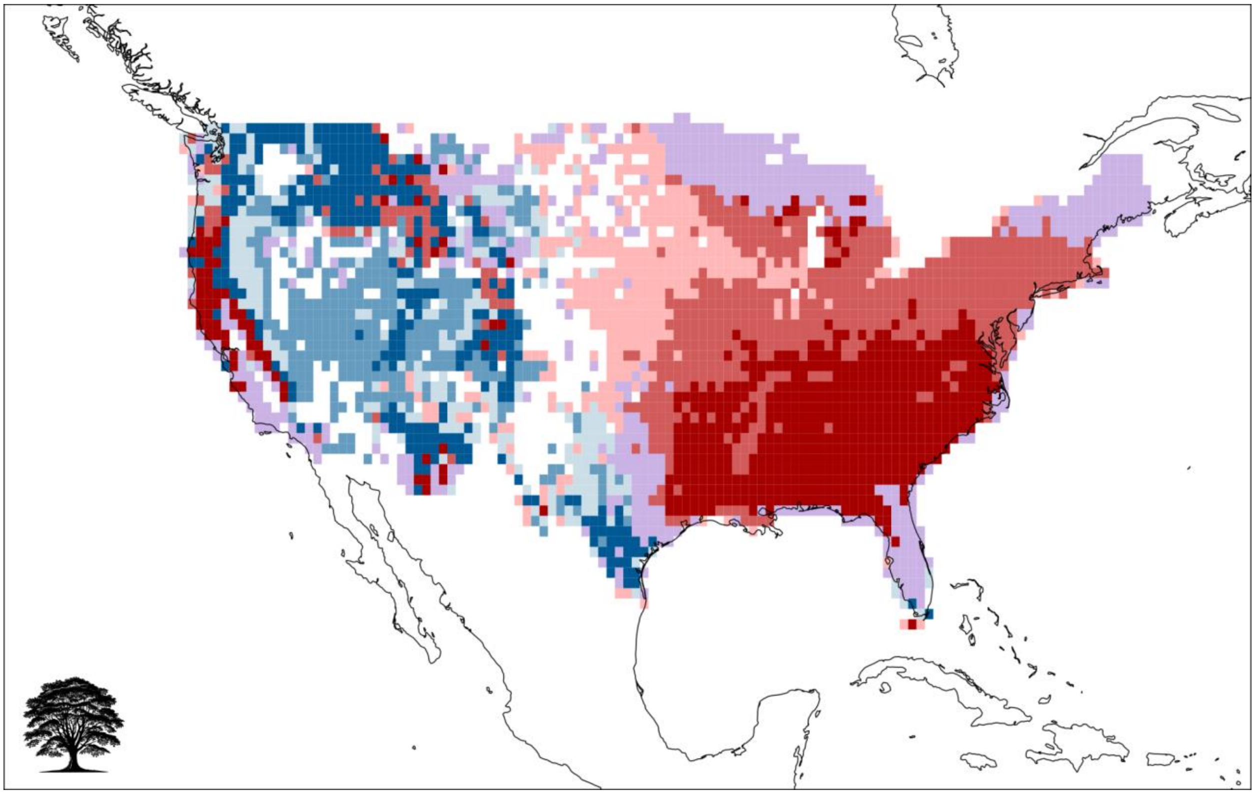

Supplementary Fig. 35 | Variance partitioning of linear models disregarding the distinct size of biogeographical regions, and thus now overrepresenting larger biogeographical regions (sensitivity analysis). Biogeographical regions with fewer than 30 grid cells were excluded to ensure model robustness. Except for amphibians and trees, the results remained qualitatively consistent with those obtained from weighted linear regressions, where equal importance was assigned to all biogeographical regions regardless of their size.

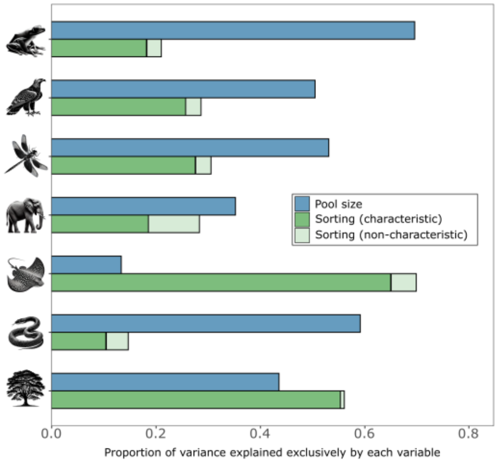

Supplementary Table 1 | Correlation values of the four-biodiversity metrics for the grid cells of the seven taxa. Upper triangular matrix shows the Pearson's r coefficient, while the lower triangular matrix the Spearman's rho rank coefficient. See Extended Data Tables 2-8 for taxon-specific values.

**Global**	Biota overlap	Species richness	Endemicity	Occupancy
Biota overlap	-	-0.15	-0.51	-0.13
Species richness	-0.13	-	0.21	-0.48
Endemicity	-0.42	0.25	-	-0.07
Occupancy	-0.14	-0.49	-0.09	-

Supplementary Table 2 | Correlation values of the four-biodiversity metrics for amphibians. Upper triangular matrix shows the Pearson's r coefficient, while the lower triangular matrix the Spearman's rho rank coefficient.

**Amphibians**	Biota overlap	Species richness	Endemicity	Occupancy
Biota overlap	-	-0.14	-0.36	-0.06
Species richness	-0.07	-	0.24	-0.59
Endemicity	-0.27	0.25	-	-0.08
Occupancy	-0.07	-0.62	-0.08	-

Supplementary Table 3 | Correlation values of the four-biodiversity metrics for birds. Upper triangular matrix shows the Pearson's r coefficient, while the lower triangular matrix the Spearman's rho rank coefficient.

**Birds**	Biota overlap	Species richness	Endemicity	Occupancy
Biota overlap	-	-0.21	-0.62	-0.03
Species richness	-0.28	-	0.11	-0.29
Endemicity	-0.44	0.23	-	-0.12
Occupancy	-0.09	-0.3	0.10	-

Supplementary Table 4 | Correlation values of the four-biodiversity metrics for dragonflies. Upper triangular matrix shows the Pearson's r coefficient, while the lower triangular matrix the Spearman's rho rank coefficient.

**Dragonflies**	Biota overlap	Species richness	Endemicity	Occupancy
Biota overlap	-	-0.05	-0.28	-0.18
Species richness	0.04	-	0.29	-0.65
Endemicity	-0.24	0.61	-	-0.13
Occupancy	-0.12	-0.72	-0.56	-

Supplementary Table 5 | Correlation values of the four-biodiversity metrics for mammals. Upper triangular matrix shows the Pearson's r coefficient, while the lower triangular matrix the Spearman's rho rank coefficient.

**Mammals**	**Biota overlap**	**Species richness**	**Endemicity**	**Occupancy**
Biota overlap	-	-0.18	-0.59	-0.13
Species richness	-0.18	-	0.17	-0.53
Endemicity	-0.52	0.12	-	0.04
Occupancy	-0.12	-0.54	0	-

Supplementary Table 6 | Correlation values of the four-biodiversity metrics for rays. Upper triangular matrix shows the Pearson's r coefficient, while the lower triangular matrix the Spearman's rho rank coefficient.

**Rays**	Biota overlap	Species richness	Endemicity	Occupancy
Biota overlap	-	0.06	-0.11	-0.31
Species richness	0.16	-	0.33	-0.59
Endemicity	0.01	0.38	-	-0.19
Occupancy	-0.35	-0.66	-0.26	-

Supplementary Table 7 | Correlation values of the four-biodiversity metrics for reptiles. Upper triangular matrix shows the Pearson's r coefficient, while the lower triangular matrix the Spearman's rho rank coefficient.

**Reptiles**	Biota overlap	Species richness	Endemicity	Occupancy
Biota overlap	-	-0.19	-0.58	-0.3
Species richness	-0.13	-	0.32	-0.48
Endemicity	-0.47	0.35	-	-0.11
Occupancy	-0.33	-0.5	-0.15	-

Supplementary Table 8 | Correlation values of the four-biodiversity metrics for trees. Upper triangular matrix shows the Pearson's r coefficient, while the lower triangular matrix the Spearman's rho rank coefficient.

**Trees**	Biota overlap	Species richness	Endemicity	Occupancy
Biota overlap	-	0.01	-0.59	0.03
Species richness	0.02	-	0.11	-0.3
Endemicity	-0.43	0.26	-	0
Occupancy	-0.03	-0.29	-0.13	-

Supplementary Table 9 | Neighbour Analysis. The area below the diagonal shows the total number of biogeographical regions, across the seven taxa, where the two compared biogeographical sectors were represented (i.e., at least one grid cell from each of the two sectors). The area above the diagonal displays the proportion of those biogeographical regions where neighbouring between pairs of sectors was higher than expected (expected probability = 1/6). Statistically significant adjacencies between sectors were identified using bidirectional binomial proportion tests for each pair of sectors (A→B and B→A), comparing observed neighbouring frequencies to expected probabilities under random distribution ($p < 0.05$). Fig. 1C shows the relative frequency of statistically significant neighbouring events between pairs of biogeographical sectors across all bioregions and taxa, normalized by the total number of significant events.

	-	0.84	0.06	0.25	0.00	0.00	0.57
	83	-	0.71	0.69	0.05	0.04	0.24
	54	55	-	0.58	0.42	0.00	0.02
	95	81	53	-	0.55	0.16	0.41
	94	75	50	86	-	0.45	0.54
	31	25	12	31	44	-	0.43
	92	70	48	90	93	46	-

Taxa	N Clusters = 2		N Clusters = 3		N Clusters = 4		N Clusters = 5		N Clusters = 6		N Clusters = 7		N Clusters = 8	
	Prop. Bioregions with signal (Total)	R^2 Mean ± SD	Prop. Bioregions with signal (Total)	R^2 Mean ± SD	Prop. Bioregions with signal (Total)	R^2 Mean ± SD	Prop. Bioregions with signal (Total)	R^2 Mean ± SD	Prop. Bioregions with signal (Total)	R^2 Mean ± SD	Prop. Bioregions with signal (Total)	R^2 Mean ± SD	Prop. Bioregions with signal (Total)	R^2 Mean ± SD
**Amphibians**	91% (34)	0.29±0.2	93% (29)	0.31±0.22	100% (26)	0.3±0.2	100% (27)	0.33±0.22	96% (23)	0.32±0.19	100% (22)	0.36±0.18	95% (20)	0.36±0.19
**Dragonflies**	100% (4)	0.25±0.13	100% (4)	0.24±0.06	100% (4)	0.31±0.1	100% (3)	0.25±0.03	100% (5)	0.22±0.08	83% (6)	0.23±0.08	100% (5)	0.31±0.17
**Birds**	100% (12)	0.28±0.15	100% (13)	0.34±0.21	100% (13)	0.32±0.21	100% (12)	0.27±0.23	100% (12)	0.27±0.17	100% (12)	0.3±0.17	100% (10)	0.27±0.11
**Mammals**	89% (19)	0.32±0.2	100% (20)	0.36±0.15	100% (17)	0.32±0.17	100% (18)	0.36±0.17	100% (20)	0.29±0.15	100% (18)	0.32±0.14	100% (16)	0.3±0.14
**Reptiles**	100% (26)	0.3±0.18	100% (27)	0.34±0.18	100% (26)	0.31±0.18	100% (24)	0.34±0.16	100% (23)	0.34±0.17	100% (24)	0.35±0.2	100% (23)	0.34±0.17
**Rays**	80% (5)	0.25±0.16	60% (5)	0.25±0.16	67% (3)	0.16±0.06	100% (2)	0.13±0.06	100% (1)	0.21	100% (1)	0.21	(0)	No data
**Trees**	100% (5)	0.31±0.18	100% (4)	0.23±0.06	100% (4)	0.2±0.06	100% (4)	0.19±0.11	75% (4)	0.22±0.08	100% (4)	0.17±0.09	100% (4)	0.19±0.09

Table S10 | Multinomial sensitivity analyses. **Prop. Bioregions with signal (Total)**: Proportion of biogeographical regions where environmental conditions statistically differ across distinct biogeographical sectors. The number in parentheses indicates the total number of bioregions assessed. This number varies across clusters because the evaluation only includes biogeographical regions that meet the following criteria: they must have at least two distinct biogeographical sectors, each sector must contain a minimum of 16 grid cells, and the combined sectors must cover at least 90% of the biogeographical region. **R^2 mean ± SD**: mean and standard deviation of McFadden's R^2 from the multinomial model applied to each biogeographical region. **N clusters**: number of biogeographical sectors selected when clustering grid cells of the seven taxa together in the k-means clustering, using the four biodiversity aspects as features (see Methods).

Taxa	N Clusters = 2		N Clusters = 3		N Clusters = 4		N Clusters = 5		N Clusters = 6		N Clusters = 7		N Clusters = 8	
	Char. Mean ± SD (N bior.)	Non-char. Mean ± SD (N bior.)	Char. Mean ± SD (N bior.)	Non-char. Mean ± SD (N bior.)	Char. Mean ± SD (N bior.)	Non-char. Mean ± SD (N bior.)	Char. Mean ± SD (N bior.)	Non-char. Mean ± SD (N bior.)	Char. Mean ± SD (N bior.)	Non-char. Mean ± SD (N bior.)	Char. Mean ± SD (N bior.)	Non-char. Mean ± SD (N bior.)	Char. Mean ± SD (N bior.)	Non-char. Mean ± SD (N bior.)
**Amphibians**	0.69±0.31 (89)	0.68±0.27 (87)	0.63±0.32 (90)	0.7±0.26 (86)	0.62±0.28 (90)	0.7±0.28 (87)	0.61±0.27 (91)	0.73±0.23 (87)	0.6±0.24 (92)	0.7±0.25 (87)	0.61±0.26 (94)	0.68±0.26 (89)	0.56±0.24 (92)	0.71±0.22 (88)
**Dragonflies**	0.69±0.38 (10)	0.53±0.27 (10)	0.51±0.4 (8)	0.57±0.18 (7)	0.7±0.28 (8)	0.6±0.32 (8)	0.58±0.39 (8)	0.5±0.26 (8)	0.6±0.35 (9)	0.53±0.21 (9)	0.84±0.22 (11)	0.58±0.3 (11)	0.6±0.41 (11)	0.62±0.21 (11)
**Birds**	0.53±0.37 (19)	0.58±0.23 (19)	0.67±0.29 (18)	0.67±0.26 (18)	0.75±0.19 (16)	0.63±0.24 (16)	0.73±0.18 (17)	0.7±0.18 (17)	0.63±0.25 (18)	0.65±0.15 (18)	0.67±0.15 (18)	0.69±0.14 (18)	0.55±0.27 (19)	0.68±0.13 (19)
**Trees**	0.48±0.5 (9)	0.72±0.3 (8)	0.8±0.36 (8)	0.64±0.18 (6)	0.81±0.18 (7)	0.55±0.33 (7)	0.83±0.14 (7)	0.56±0.24 (7)	0.78±0.17 (8)	0.65±0.26 (8)	0.78±0.22 (9)	0.57±0.24 (8)	0.76±0.2 (9)	0.65±0.27 (8)
**Mammals**	0.74±0.29 (35)	0.79±0.26 (34)	0.71±0.24 (33)	0.8±0.24 (30)	0.65±0.26 (36)	0.77±0.22 (33)	0.61±0.23 (37)	0.8±0.22 (34)	0.64±0.23 (37)	0.7±0.23 (34)	0.57±0.21 (36)	0.69±0.21 (33)	0.56±0.21 (37)	0.71±0.24 (34)
**Rays**	0.81±0.35 (16)	0.64±0.25 (12)	0.75±0.34 (17)	0.68±0.34 (13)	0.76±0.28 (17)	0.67±0.34 (14)	0.75±0.28 (18)	0.72±0.28 (14)	0.75±0.23 (21)	0.7±0.33 (17)	0.82±0.21 (20)	0.76±0.25 (16)	0.74±0.25 (21)	0.7±0.32 (17)
**Reptiles**	0.61±0.33 (55)	0.76±0.27 (54)	0.67±0.25 (54)	0.8±0.23 (49)	0.66±0.25 (57)	0.77±0.19 (53)	0.56±0.29 (62)	0.78±0.2 (58)	0.59±0.3 (69)	0.72±0.24 (65)	0.58±0.26 (62)	0.75±0.2 (58)	0.54±0.28 (69)	0.71±0.24 (65)

Table S11 | Nestedness sensitivity analyses. Predominance of a nestedness pattern over a turnover one in the dissimilarity of the characteristic (Char.) and non-characteristic (Non-char.) biota across biogeographical sectors within each biogeographical region. The number in parenthesis represent the number of biogeographical sectors evaluated. This number varies across clusters because the evaluation only includes biogeographical regions that meet the following criteria: they must have at least two distinct biogeographical sectors with any number of grid cells. **Mean ± SD**: mean and standard deviation, across biogeographical regions, of the proportion of the biotic dissimilarity across biogeographical sectors attributed to nestedness. This proportion was calculated as the ratio of the nestedness component and the total Sørensen dissimilarity (nestedness + turnover). See details in Methods. **N clusters**: number of biogeographical sectors selected when clustering grid cells of the seven taxa together in the k-means clustering, using the four-biodiversity metrics as features (see Methods).

Supplementary Table 12 | Optimal number of biogeographical sectors clustering grid cells for each taxon. We used these taxon-specific biogeographical sectors to search for the general sectors—delineated by clustering grid cells of the seven taxa together—that represent all the taxa the best. By using this approach, we found an optimum of seven general biogeographical sectors. However, sensitivity analyses repeating all our analyses using from two to eight general biogeographical sectors (see main text) demonstrated that our results and inferences are robust to the choice of the number of sectors (Supplementary Figs. S8-13 and Supplementary Tables S10 and 11).

**Taxon**	**Optimal number of clusters using taxon-data individually**
Amphibians	8
Birds	7
Dragonflies	6
Mammals	6
Rays	8
Reptiles	7
Trees	7

Appendix A | Correlation between current biodiversity and past climatic conditions

We conducted additional analyses for terrestrial biota to assess the correlation of the core-to-transition pattern with present climate conditions and climate change since the Last Glacial Maximum (LGM; 21ka). For each taxon and biogeographical region, we repeated the multinomial model incorporating four explanatory variables: present mean annual temperature and precipitation, alongside the difference in the average present and past (LGM) temperature and precipitation[83], both modelled across eight climatic model[84]. Evidence presented in the main text was considered statistically significant when the corrected Akaike Information Criterion (ΔAICc) for the model including any of the present and past climatic factors was at least 10 units lower than that of the intercept-only model[74]. Additionally, we explored the "apparent" relative importance of present climate versus climate change since the LGM by calculating the difference between the non-shared variance explained by the present and climate change conditions, divided by the total variance explained by both variables together (i.e. sum of shared and non-shared explained variance). Values greater than 0 indicate that the studied present climate conditions were more strongly correlated with the biogeographical sectors, whereas values below 0 indicate stronger correlations with climate change since the LGM (Supplementary Figs. S14-26). The relative importance of present and past climate is based on a limited set of plausible environmental factors (temperature and precipitation) and results can vary when considering other variables and studied scale[80]. Therefore, our inferences in the main text focus primarily on the existence of significant signals of present and past climatic conditions, as indicated by the ΔAICc.

Appendix B | Robustness of bioregion delineations

To evaluate the robustness of Infomap in delineating biogeographical regions, we conducted a sensitivity analysis comparing its bioregions with those produced by the Stochastic Block Model (SBM)[63], an established alternative in network science for grouping nodes. We used the graph-tool library[85] (version 2.31). While Infomap capitalizes on coding theory, the Stochastic Block Model (SBM) employs probabilistic inference. More relevant to this robustness analysis is how they cluster grid cells and species: Infomap jointly groups them so that probability flow along links persists within well-connected biogeographical regions, whereas SBM clusters them separately based on similarities in their connection patterns to other nodes. By comparing these approaches, we aimed to assess whether clustering based on distinct connectivity patterns yield similar biogeographical regions.

Using the Infomap partition presented in our main text as a reference, we identified the most comparable hierarchical level in the SBM output. We quantified the similarity between Infomap and SBM partitions using the Adjusted Mutual Information (AMI), a widely used metric in network science for objective comparison of partitions. Our results showed high AMI values (overall, 0.75 ± 0.03; mean ± standard error. For amphibians = 0.79, birds = 0.83, dragonflies = 0.69, mammals = 0.76, rays = 0.76, reptiles = 0.79, and trees = 0.66). These findings indicate strong agreement between the biogeographical regions identified by Infomap and the SBM.

This comparison between Infomap and SBM contributes to the broader literature by demonstrating that Infomap produces bioregions similar to—or in some cases, even more refined than—those obtained using alternative well-established biogeographical methods, such as agglomerative hierarchical clustering and modularity[25,52,59]. This congruence, alongside evidence showing their accuracy in representing species' distribution range overlaps[25], support the robustness of Infomap's bioregion delineation. This reinforces its validity for addressing broader applications, including assessing general biodiversity patterns within biogeographical regions across terrestrial and marine vertebrates, invertebrates, and plants.

Appendix C | K-means clustering controlling for the distinct number of grid cells per taxon

We conducted an additional sensitivity analysis to account for variations in the number of grid cells per taxon. This sensitivity analysis provided an equal representation for each taxon, but indirectly increasing the representativeness of Eurasia and North America, among other areas, due to the continental information of dragonflies and trees. To maintain consistency in the k-means analyses in both main and sensitivity analyses, we used in both cases the function *kmeans.weight* of the R package SWKM[74]. In our main analyses, we established a similar weight to all observations, by weighting each observation as one divided by the total number of grid cells across taxa. In the sensitivity analyses, we weighted each observation as the inverse of the number of grids of its taxon. We obtained the same qualitative results in both main and sensitivity analyses (Extended Data Figs. 2-8, Supplementary Figs. 28-34). These results support that the biogeographical sectors identified are not inherently linked to specific taxa or biogeographical regions but rather exhibit a more general nature.

Appendix D | Delineating biogeographical regions and estimating biodiversity aspects from data with distinct geographical extent

The extent of the dataset can influence the quantity of information analyzed and, consequently, the resolution limits of community detection algorithms, such as their capacity to detect fine-scale communities. In biogeographical terms, delineating regions at varying extents (e.g., global or continental) can affect the maximum resolution of the regions detected, or the lowest hierarchical level of biodiversity organization (e.g., realms, regions, dominions, provinces, and districts[58]. However, Infomap is relatively robust to issues with the resolution limit[24] and the obtained biogeographical delineations are minimally affected (see below). On the other hand, limiting the delineation of biogeographical regions to continental extents can introduce potential biases in the classification of species into characteristic and non-characteristic. In particular, species whose primary distributions lie outside the studied area may be erroneously classified as characteristic of a biogeographical region within the studied area. Such misclassifications can affect the estimation of the biodiversity aspects, particularly when defining regional hotspots or identifying transitional areas. However, the impact of this issue is expected to be minor, given that relatively few species have transcontinental distributions.

To empirically demonstrate that our results are not affected by considering different geographical extents, we conducted a sensitivity analysis focusing on five taxa with global datasets: amphibians, birds, mammals, rays, and reptiles. For these taxa with data at global extent, we delineated the biogeographical regions and estimated the biodiversity metrics using their data at ca. continental or biogeographical realm extent independently. For each taxon, we first identified the largest hierarchical level corresponding to biogeographical realms[19]. These realms were treated as independent datasets for subsequent analyses. Within each realm, we applied the same methodologies used for the global datasets to delineate biogeographical regions and calculate biodiversity metrics. This allowed for a direct comparison between the regions and metrics derived from the global datasets and those derived from the smaller, realm-specific datasets.

To compare bioregions, we calculated Adjusted Mutual Information[73] (AMI) values between regions identified using global datasets and those identified using realm-based datasets. For

biodiversity metrics, we used Spearman's correlations to assess the similarity of metric values between the two dataset types. The comparisons revealed high consistency in both biogeographical regions and biodiversity metrics, regardless of the geographic extent of the datasets. AMI values were very close to 1 (mean ± standard deviation = 0.94 ± 0.03), indicating strong agreement between the bioregions derived from global datasets and those derived from realm-based datasets. Similarly, biodiversity metrics showed very high positive correlations across the two dataset types, with Spearman's rho values averaging 0.83 ± 0.08.

These results demonstrate that the biogeographical regions and biodiversity metrics identified are robust to variations in the geographic coverage of the datasets. This robustness supports the validity of using either global or continental datasets to define bioregions and calculate biodiversity metrics, providing confidence in the broader applicability of our approach.

Appendix E | Residual spatial autocorrelation

Spatial autocorrelation in species distributions and environmental variables is a natural phenomenon and does not pose a challenge when evaluating relationships among variables[86-88]. However, spatial autocorrelation in residuals indicates a lack of independence among observations, which can lead to several issues: underestimation of standard errors, inflation of Type I errors (i.e., overestimation of statistically significant results), and misrepresentation of the relative importance of variables[87-91]. The risk is to obtain spurious correlations and then wrong inferences.

To evaluate spatial autocorrelation in residuals, residuals must be calculated from the fitted model. However, to our knowledge, there is no established approach for estimating residuals in non-ordinal multinomial models where each observation is fully assigned to one category, such as assigning grid cells to a single biogeographical sector. Given that multinomial models are an extension of logistic regression, we performed sensitivity analyses using pairwise logistic regressions between biogeographical sectors within each bioregion. In these logistic regressions, the dependent variable (coded as 1 or 0) represents the two biogeographical sectors being compared, while the explanatory variables are the same environmental factors used in the multinomial models. This approach allows us to estimate residuals and test for spatial autocorrelation (Moran's test, p-value < 0.05). When residual spatial autocorrelation was detected (in at least one pairwise comparison in 85 out of 87 of biogeographical regions across all taxa), we incorporated in the logistic regression a spatial component using a Matérn covariance function[92]. The Matérn covariance function is a flexible way to model spatial relationships because it has a "smoothness parameter" that can adapt the spatial structure to the specific data being analyzed[92]. It generalizes other commonly used functions, such as Gaussian or exponential functions, allowing it to capture a wider range of spatial patterns[92]. After confirming that residual spatial autocorrelation was addressed in the saturated spatial models, containing our environmental variables plus the Matérn covariance function, we assessed for the presence of a climatic signal. Specifically, we compared the AICc of the spatial model with the one of a null model including only the same spatial structure. To fix the spatial structure in the null model and make the saturated and null model comparable, we extracted the parameters rho, nu, and lambda from the saturated model (i.e., parameters defining the spatial structure), and used them to fix the rho, nu and lambda parameters in the null model, which only include the Matérn covariance function. We considered

the existence of an environmental signal, if any saturated model fitted for a biogeographical region, which include our explanatory variables and the spatial matrix, had a ΔAICc of -10 or lower compared to the null spatial model[79]. Our results show an environmental signal in all biogeographical regions, except in one for birds. These analyses provide conservative estimates, as only two environmental variables were considered, and additional factors could reveal more and stronger associations.

References

83. Calatayud, J. et al. Glaciations, deciduous forests, water availability and current geographical patterns in the diversity of European Carabus species. J. Biogeogr. 43, 2343-2353 (2016).
84. Lima-Ribeiro, M. S. et al. EcoClimate: a database of climate data from multiple models for past, present, and future for macroecologists and biogeographers. Biodiversity Informatics 10, (2015).
85. Peixoto, T.P. "The graph-tool python library", figshare. (2014) DOI: 10.6084/m9.figshare.1164194 [sci-hub, @tor].
86. Hawkins, B. A. Eight (and a half) deadly sins of spatial analysis. J. Biogeogr. 39, 1–9 (2012).
87. Diniz-Filho, J. A. F., Bini, L. M. & Hawkins, B. A. Spatial autocorrelation and red herrings in geographical ecology. Glob. Ecol. Biogeogr. 12, 53–64 (2003). https://doi.org/10.1046/j.1466-822X.2003.00322.x
88. Kühn, I. & Dormann, C. F. Less than eight (and a half) misconceptions of spatial analysis. J. Biogeogr. 39, 995–998 (2012). https://doi.org/10.1111/j.1365-2699.2012.02734.x
89. Legendre, P. Spatial autocorrelation: trouble or new paradigm? Ecology 74, 1659–1673 (1993). https://doi.org/10.2307/1939924
90. Diniz-Filho, J. A. F., Hawkins, B. A., Bini, L. M., Blackburn, T. M. & De Marco, P. Are spatial regression methods a panacea or a Pandora's box? A reply to Beale et al. (2007). Ecography 30, 848–851 (2007). https://doi.org/10.1111/j.2007.0906-7590.05358.x
91. Bini, L. M. et al. Coefficient shifts in geographical ecology: an empirical evaluation of spatial and non-spatial regression. Ecography 32, 193–204 (2009). https://doi.org/10.1111/j.1600-0587.2009.05717.x
92. Wang, K., Abdulah, S., Sun, Y. & Genton, M. G. Which parameterization of the Matérn covariance function? Spat. Stat. 58, 100787 (2023). https://doi.org/10.1016/j.spasta.2023.100787
