# Supplementary material for: A general rule on the organization of biodiversity in Earth’s biogeographical regions
Source: Nat Ecol Evol. 2025 Jun 4;9(7):1193–204. doi: 10.1038/s41559-025-02724-5 (PMC12240819; doi:10.1038/s41559-025-02724-5)
Supplement: Supplementary file 2 — Reporting Summary [file 41559_2025_2724_MOESM2_ESM.pdf]

nature portfolio

Corresponding author(s): Rubén Bernardo Madrid
ruben.bernardo.madrid@umu.se

Last updated by author(s): Apr 9, 2025

Reporting Summary

Nature Portfolio wishes to improve the reproducibility of the work that we publish. This form provides structure for consistency and transparency in reporting. For further information on Nature Portfolio policies, see our Editorial Policies and the Editorial Policy Checklist.

Statistics

For all statistical analyses, confirm that the following items are present in the figure legend, table legend, main text, or Methods section.

n/a	Confirmed	
☐	☒	The exact sample size (*n*) for each experimental group/condition, given as a discrete number and unit of measurement
☒	☐	A statement on whether measurements were taken from distinct samples or whether the same sample was measured repeatedly
☐	☒	The statistical test(s) used AND whether they are one- or two-sided<br>*Only common tests should be described solely by name; describe more complex techniques in the Methods section.*
☐	☒	A description of all covariates tested
☒	☐	A description of any assumptions or corrections, such as tests of normality and adjustment for multiple comparisons
☐	☒	A full description of the statistical parameters including central tendency (e.g. means) or other basic estimates (e.g. regression coefficient) AND variation (e.g. standard deviation) or associated estimates of uncertainty (e.g. confidence intervals)
☒	☐	For null hypothesis testing, the test statistic (e.g. *F*, *t*, *r*) with confidence intervals, effect sizes, degrees of freedom and *P* value noted<br>*Give P values as exact values whenever suitable.*
☒	☐	For Bayesian analysis, information on the choice of priors and Markov chain Monte Carlo settings
☒	☐	For hierarchical and complex designs, identification of the appropriate level for tests and full reporting of outcomes
☐	☒	Estimates of effect sizes (e.g. Cohen's *d*, Pearson's *r*), indicating how they were calculated

Our web collection on statistics for biologists contains articles on many of the points above.

Software and code

Policy information about availability of computer code

Data collection	Data used is provided free of charge by IUCN, BirdLife, and Forest Inventory of United States (FIA)
Data analysis	All analyses were run in R software (Version 4.3.0). R code and objects are provided in the SI<br>R package SWKM version 0.09<br>R package aricode function AMI version 1.0.3<br>R package nnet Version 7.3-18<br>R package nnet Version 0.99.51<br>R package betapart Version 1.6<br><br>Infomap https://www.mapequation.org/infomap/ Version 2.6.1

For manuscripts utilizing custom algorithms or software that are central to the research but not yet described in published literature, software must be made available to editors and reviewers. We strongly encourage code deposition in a community repository (e.g. GitHub). See the Nature Portfolio guidelines for submitting code & software for further information.

Data

Policy information about availability of data

All manuscripts must include a data availability statement. This statement should provide the following information, where applicable:

- Accession codes, unique identifiers, or web links for publicly available datasets
- A description of any restrictions on data availability
- For clinical datasets or third party data, please ensure that the statement adheres to our policy

The data supporting the findings of this study are publicly available from established repositories. Species distribution maps for amphibians, mammals, reptiles, rays, and dragonflies were obtained from the International Union for Conservation of Nature (IUCN; https://www.iucnredlist.org). Bird species distributions were sourced from BirdLife International (https://www.birdlife.org), and tree occurrence data from the United States Forest Inventory and Analysis Program (FIA; https://www.fia.fs.usda.gov). Climate data used in this study were obtained from the CRU-TS 4.06 and WorldClim 2.1 databases for terrestrial taxa, and from NASA's Ocean Color and NOAA's World Ocean Atlas 2009 for marine taxa. All raw distribution data are freely accessible for academic use upon request from the respective repositories or via their websites.

Research involving human participants, their data, or biological material

Policy information about studies with human participants or human data. See also policy information about sex, gender (identity/presentation), and sexual orientation and race, ethnicity and racism.

Reporting on sex and gender	not apply
Reporting on race, ethnicity, or other socially relevant groupings	not apply
Population characteristics	not apply
Recruitment	not apply
Ethics oversight	not apply

Note that full information on the approval of the study protocol must also be provided in the manuscript.

Field-specific reporting

Please select the one below that is the best fit for your research. If you are not sure, read the appropriate sections before making your selection.

☐ Life sciences ☐ Behavioural & social sciences ☒ Ecological, evolutionary & environmental sciences

For a reference copy of the document with all sections, see nature.com/documents/nr-reporting-summary-flat.pdf

Ecological, evolutionary & environmental sciences study design

All studies must disclose on these points even when the disclosure is negative.

Study description	Analyses on biodiversity patterns based on distribution range maps of amphibians, birds, dragonflies, mammals, rays, and reptiles, as well as forest inventory of United States
Research sample	30,049 marine and terrestrial vertebrates, invertebrates and plant species
Sampling strategy	Not apply
Data collection	Downloaded from IUCN, BirdLife and FIA websites
Timing and spatial scale	Data downloaded in 2021. The extend of the data is planetary
Data exclusions	not apply
Reproducibility	Freely available data and R code provided
Randomization	not apply
Blinding	not apply

Did the study involve field work? ☐ Yes ☒ No

Reporting for specific materials, systems and methods

We require information from authors about some types of materials, experimental systems and methods used in many studies. Here, indicate whether each material, system or method listed is relevant to your study. If you are not sure if a list item applies to your research, read the appropriate section before selecting a response.

Materials & experimental systems

n/a	Involved in the study
☒	☐ Antibodies
☒	☐ Eukaryotic cell lines
☒	☐ Palaeontology and archaeology
☒	☐ Animals and other organisms
☒	☐ Clinical data
☒	☐ Dual use research of concern
☒	☐ Plants

Methods

n/a	Involved in the study
☒	☐ ChIP-seq
☒	☐ Flow cytometry
☒	☐ MRI-based neuroimaging

Plants

Seed stocks	not apply
Novel plant genotypes	not apply
Authentication	not apply
